# Acute Blood Pressure Lowering and Risk of Ischemic Lesions on MRI After Intracerebral Hemorrhage

**DOI:** 10.1001/jamaneurol.2025.0586

**Published:** 2025-04-21

**Authors:** Ken S. Butcher, Brian Buck, Dar Dowlatshahi, Laura C. Gioia, Mahesh Kate, Ana C. Klahr, Ashwin Sivasubramaniam, Ashfaq Shuaib, Alan Wilman, Vijay K. Sharma, Georgios Tsivgoulis, Christos Krogias, Ashkan Shoamanesh

**Affiliations:** 1 School of Clinical Medicine, University of New South Wales, Sydney, Australia; 2 Division of Neurology, University of Alberta, Edmonton, Alberta, Canada; 3 Division of Neurology, University of Ottawa, Ottawa, Ontario, Canada; 4 Ottawa Hospital Research Institute, Ottawa, Ontario, Canada; 5 Department of Neuroscience, Centre de recherche du Centre hospitalier de l’Université de Montréal, Montréal, Quebec, Canada; 6 Department of Social Sciences, Augustana Faculty, University of Alberta, Camrose, Alberta, Canada; 7 Department of Biomedical Engineering, University of Alberta, Edmonton, Alberta, Canada; 8 Yong Loo Lin School of Medicine, National University of Singapore and National University Hospital; 9 Second Department of Neurology, School of Medicine, National and Kapodistrian University of Athens, Attikon University General Hospital, Athens, Greece; 10 Department of Neurology, EvK Herne, Academic Teaching Hospital of the Ruhr, University of Bochum, Herne, Germany; 11 Population Health Research Institute, McMaster University, Hamilton, Ontario, Canada

## Abstract

**Importance:**

Diffusion-weighted imaging (DWI) lesions have been demonstrated in patients with subacute intracerebral hemorrhage (ICH), suggesting ischemic injury, which may be related to blood pressure (BP) reduction.

**Objective:**

To test the hypothesis that acute intensive BP lowering is associated with DWI lesions after ICH.

**Design, Setting, and Participants:**

The Intracerebral Hemorrhage Acutely Decreasing Arterial Pressure Trial 2 (ICHADAPT-2) was a multicenter, randomized, open-label, blinded–end point trial. Between November 2012 and August 2022, patients with ICH presenting within 6 hours of onset were randomized to a systolic BP (SBP) target of less than 140 mm Hg or less than 180 mm Hg. The trial was conducted at 3 comprehensive stroke centers in Canada and Australia, including 1 telestroke referral hub and 1 community stroke hospital. A total of 162 patients with acute ICH were randomized. The primary analysis population was restricted to those undergoing DWI at 48 hours.

**Intervention:**

Patients were randomly assigned to an acute SBP target of less than 140 mm Hg or less than 180 mm Hg.

**Main Outcome and Measure:**

The primary end point was the incidence of acute DWI lesions on brain magnetic resonance imaging obtained 48 ± 12 hours after randomization.

**Results:**

DWI was obtained in 79 (48% female) patients with a mean (SD) age of 71 (13) years and median baseline ICH volume of 11.2 (range, 0.5-122.2) mL. The median times from onset to randomization and DWI were 3.17 (range, 0.7-14.6) hours and 51.6 (range, 17.0-121.4) hours, respectively. Mean (SD) baseline SBP was 183 (22) mm Hg in the less than 140 mm Hg target group and 181 (28) mm Hg in the less than 180 mm Hg target group. Mean SBP was lower over the 48-hour period after randomization in the less than 140 mm Hg group (mean difference, 18.9 mm Hg [95% CI, 17.6-20.2]; *P* < .001). DWI lesions were detected in 13 of 42 patients (31%) in the less than 140 mm Hg group and 14 of 37 patients (38%) in the less than 180 mm Hg group (odds ratio, 0.74 [95% CI, 0.12-4.64]; *P* = .32). The median number of DWI lesions (1 [95% CI, 1-10] vs 1.5 [95% CI, 1-10]; *P* = .26) and total DWI lesion volume (0.1 [95% CI, 0.01-41.3] mL vs 0.3 [95% CI, 0.02-2.03] mL; *P* = .17) were not different in the less than 140 mm Hg and less than 180 mm Hg groups.

**Conclusions and Relevance:**

DWI lesion frequency and volume were unaffected by intensive antihypertensive therapy. These results support the safety of early BP reduction in acute ICH.

## Key Points

**Question:**

Does acute blood pressure (BP) reduction increase the incidence of subacute ischemic lesions in spontaneous intracerebral hemorrhage (ICH)?

**Findings:**

The frequency of ischemic lesions detected with diffusion-weighted magnetic resonance imaging at 48 hours did not increase in patients with ICH randomized to an intensive systolic BP target of less than 140 mm Hg within 6 hours of onset, compared with those with a target of less than 180 mm Hg.

**Meaning:**

Study findings support the safety of systolic BP reduction to a target of less than 140 mm Hg in patients with acute ICH.

## Teaser

This multicenter, randomized clinical trial examines whether systolic blood pressure reduction increases the incidence of lesions detected with diffusion-weighted imaging in adult patients with acute intracerebral hemorrhage.

## Introduction

Current guidelines indicate that systolic blood pressure (SBP) should be treated to a target range of 130 to 140 mm Hg in acute intracerebral hemorrhage (ICH).^1^ This intervention is supported by randomized clinical trials demonstrating intensive blood pressure (BP) reduction may improve clinical outcomes.^2,3,4^ None of these trials systematically assessed for ischemic injury with diffusion-weighted imaging (DWI), which is the optimal investigation given its sensitivity for small-volume infarcts.^5^

Cross-sectional magnetic resonance imaging (MRI) studies in acute and subacute ICH indicate that DWI lesions remote from the hematoma occur in 11% to 59% of patients with ICH.^6,7,8,9,10,11,12,13,14,15,16,17,18^ Although the etiology of these lesions is unknown, they have been hypothesized to represent ischemia, making them relevant to the management of BP in patients with acute ICH, as a drop in cerebral perfusion pressure may precipitate or aggravate ischemic injury.

Retrospective observational studies have yielded conflicting results with respect to the association between DWI lesions and BP. A pooled analysis of retrospective studies indicated that lower BP was not associated with an increased risk of DWI lesions.^19^ However, other studies demonstrated significant correlations between baseline BP and/or treatment with antihypertensive agents and DWI lesion incidence.^6,15^ In a secondary analysis of the Antihypertensive Treatment of Acute Cerebral Hemorrhage II (ATACH-2) trial, DWI lesions were not associated with intensive SBP reduction with nicardipine, although this was a post hoc analysis in a study subset.^13^ The Intracerebral Hemorrhage Acutely Decreasing Arterial Pressure Trial 2 (ICHADAPT-2) was designed to test the hypothesis that SBP reduction in acute ICH increases the incidence of DWI lesions.

## Methods

### Study Design

The ICHADAPT-2 trial was a multicenter, prospective, randomized, open-label treatment trial with blinded–end point assessment of ischemic lesion frequency, detected with DWI, after acute BP reduction. Eligible patients with ICH were randomized 1:1 to either an SBP target of less than 140 mm Hg or less than 180 mm Hg (NCT02281838).^20^ The trial protocol (Supplement 1) was approved by local human research ethics boards. Informed written consent was obtained from each patient or substitute decision maker. A deferred consent procedure was used in cases where the patient was unable to consent due to neurological disability and a surrogate decision maker was not available. In deferred consent cases, written consent was obtained prior to undergoing MRI.

### Study Population and Randomization

Eligible patients were 18 years or older with ICH 6 hours or less from onset and 2 SBP measurements of 140 mm Hg or greater. Patients with hematoma volumes greater than 90 mL estimated using the ABC/2 method^21^ or Glasgow Coma Scale (GCS) scores of 5 or less (range, 3-15; higher scores indicate greater responsiveness), a definite contraindication to BP reduction, definite indication for BP reduction, known contraindication to MRI, evidence of a macrovascular or secondary etiology of ICH, ischemic stroke within 90 days, premorbid modified Rankin Scale (mRS) score of 3 or greater (range, 0-6; higher scores indicate greater disability), or planned early surgical resection were excluded.

Patients were randomized using permuted blocks of randomly varying sizes of 4, 6, or 8, stratified by hospital site, age, GCS score, anticoagulant use, baseline ICH volume, and intraventricular extension. Only the database programmer was aware of the allocation table and all investigators and patients were masked. Study data were collected and managed using REDCap electronic data capture.^17^

### Study Procedures

Baseline BP, GCS score, National Institutes of Health Stroke Scale (NIHSS [range, 0-42; higher scores indicate more severe stroke symptoms]) and premorbid disability (mRS) scores, stroke risk factors, and medications were recorded prior to randomization. Neurological and functional disability assessments were repeated at days 2, 7, and 30.

### BP Management

Following randomization, the assigned BP target was achieved and maintained using an intravenous antihypertensive drug bolus protocol based on labetalol and hydralazine (Supplement 1). The goal of treatment was to achieve the assigned target within 1 hour of randomization and to maintain this target for 24 hours. Noninvasive monitoring of BP and heart rate was continued for a minimum of 24 hours after randomization. Missing BP values were imputed using interpolation.

### Imaging Procedures and Assessments

At 24 ± 3 hours after randomization, noncontrast computed tomography was performed to assess for hematoma expansion. At 48 ± 12 hours after randomization, patients underwent an MRI, including DWI. Repeat MRI was obtained 7 ± 2 and 30 ± 5 days after randomization. All images, including the baseline diagnostic noncontrast computed tomography scan, were analyzed by raters blinded to BP treatment. DWI lesions were defined as remote areas outside of the perihematoma border that were hyperintense on DWI with either corresponding hypointensity or isointensity on apparent diffusion coefficient maps.^13^ Lesion location and volumes were assessed using 3D Slicer.^22^

### Study Outcomes

The primary end point was DWI lesion incidence on 48-hour MRI. Secondary end points included incident lesions on repeat MRI at 7 and 30 days, median number of DWI lesions, and total DWI lesion volume.

### Statistical Analysis

The statistical analysis plan is provided in Supplement 2. The planned sample size of 270 patients included a 50% inflation due to the frequent inability to obtain an MRI following acute ICH due to medical instability. The aim was to obtain 180 evaluable patients, providing 80% power to detect a 22% increase in the frequency of DWI lesions. We assumed equal allocation (1:1) to both BP target groups with no correction for continuity. The sample size was based on a 1-sided test of independent proportions at the α = .025 level. There were no interim analyses.

The primary analysis was completed using an intention-to-treat approach, where all patients were included in the groups into which they were originally randomized, irrespective of actual achieved BP control. Differences in BP from randomization to 48 hours were assessed using a repeated-measure linear mixed model with a fixed effect of treatment group, fixed categorical effect of time, fixed interaction between treatment and time, and repeated patient effect, with adjustment for baseline measurements, as previously described.^2^

An unadjusted 2-sample comparison of proportions was used to assess the primary outcome. Other differences between groups were tested using *t* tests (continuous variables), Mann-Whitney *U* tests (non-normally distributed and ordinal variables), or Fisher exact tests (categorical variables). Predictors of DWI lesions were examined in exploratory analyses using a univariable regression analysis that compared baseline demographics and clinical characteristics between those patients with and without DWI lesions. Variables significant at the *P* < .10 level were included in a multivariable logistic regression model. All statistical tests were 2-sided with *P* < .05 considered statistically significant. A study-level meta-analysis of the effects of intensive BP reduction on DWI lesion incidence was performed by combining the results of the ATACH-2 MRI analysis with those of the current study. Statistical analyses were completed using Python (Python Software Foundation), with the NumPy, Statsmodels, and SciPy libraries.^23,24,25^ Meta-analyses were completed using RevMan version 5.4 (Cochrane).

### Data Safety Monitoring

An independent safety committee reviewed all serious adverse events after 135 patients were enrolled to ensure that neither group had a disproportionate accumulation of adverse events. No interim analyses were planned.

### Meta-Analysis, Search Strategy, and Selection Criteria

We performed a post hoc systematic review of the literature to identify previous randomized clinical trials comparing intensive vs usual BP lowering in patients with acute ICH (≤12 hours from onset) undergoing MRI within 10 days of ICH. We searched PubMed for studies published up to December 18, 2024, without language restrictions, using the search terms: *intensive blood pressure* or *<140 mm Hg*, *control* or *target*, *intracerebral hemorrhage*, *randomized* or *random* and *allocation*, *DWI*, and *lesions* or *hyperintensities* or *hyperintense*. Summary data were extracted from the identified trial. Meta-analyses were performed using a random-effects model for the outcome of incident DWI lesions to estimate risk ratios. The *I^2^* test was used to evaluate heterogeneity.

## Results

### Participant Characteristics

**Figure 1.**
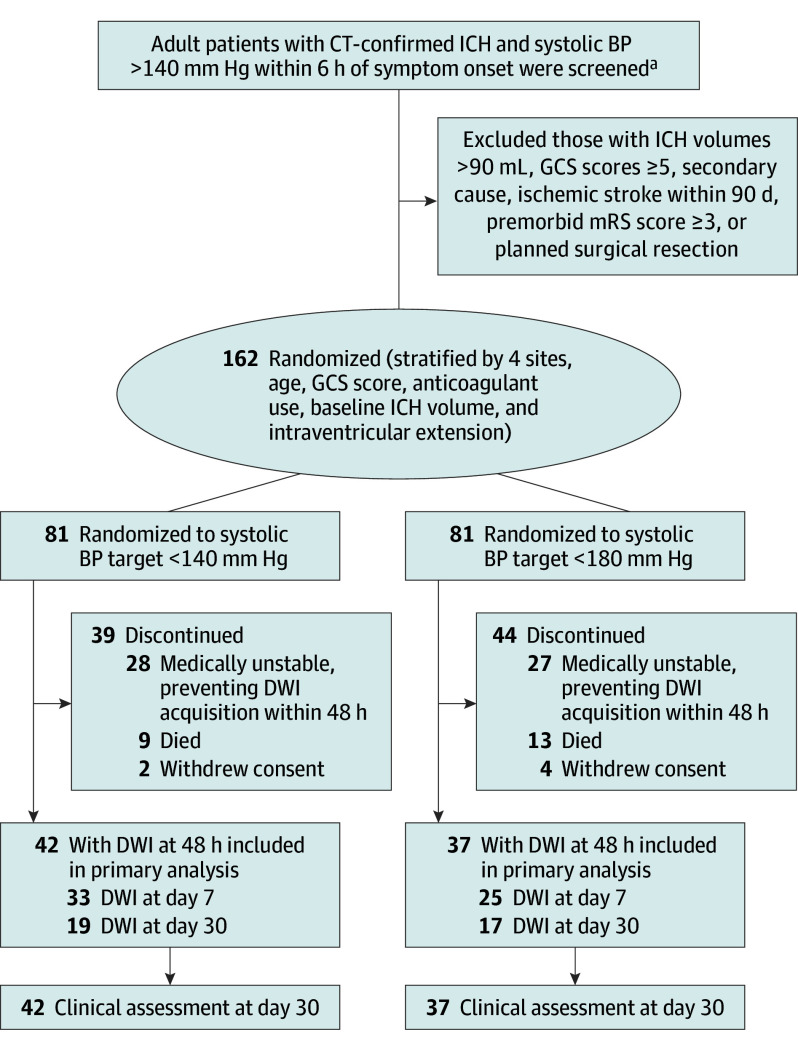
Flowchart of Randomization, Blood Pressure (BP) Target Allocation, and Follow-Up ^a^Study sites were not required to maintain screening logs, thus the total number of patients assessed for study eligibility was not available. CT indicates computed tomography; DWI, diffusion-weighted imaging; GCS, Glasgow Coma Scale; ICH, intracerebral hemorrhage; mRS, modified Rankin Scale.

Between November 2012 and August 2022, 162 patients with acute ICH were randomized at 4 sites. Following presentation and publication of the third Intensive Care Bundle With Blood Pressure Reduction in Acute Cerebral Hemorrhage Trial (INTERACT3),^2^ practice patterns at participating sites changed to include more routine use of a target SBP of less than 140 mm Hg due to a perceived loss of clinical equipoise. After consultation with the data and safety monitoring board in April 2024, the executive committee halted enrollment. Of 162 randomized patients, 79 underwent DWI at 48 hours and were included in the primary analysis (Figure 1).

The baseline characteristics of the primary analysis patients are shown in Table 1. The median time from symptom onset to randomization was 3.17 (range, 0.7-14.6) hours. DWI was obtained at a median of 51.6 (range, 17.0-121.4) hours in patients with a median ICH volume of 11.2 (range, 0.5-122.2) mL. Of the 79 patients with a DWI (primary end point), 42 were randomized to an SBP target of less than 140 mm Hg and 37 to less than 180 mm Hg. Relative to patients without MRI, those in whom a DWI was obtained had lower ICH volumes, NIHSS scores, and early mortality rates than those who did not undergo MRI (Table 2).

**Table 1.** Baseline Demographic and Intracerebral Hemorrhage (ICH) Characteristics of Primary Analysis Patients

Characteristic	Target SBP, No. (%)	
	<140 mm Hg (n = 42)	<180 mm Hg (n = 37)
Demographics		
Age, mean (SD), y	70 (12)	73 (14)
Sex		
Female	22 (52)	14 (38)
Male	20 (48)	23 (62)
Medical history		
Hypertension	31 (74)	24 (65)
Previous ICH	3 (7)	3 (8)
Previous ischemic stroke	5 (12)	7 (19)
Ischemic heart disease	2 (5)	1 (3)
Diabetes	12 (29)	7 (19)
Smoking (current)	4 (10)	3 (8)
Alcohol use (current)	8 (5)	5 (3)
Medications		
Antihypertensive agent	9 (21)	7 (19)
Anticoagulant	5 (12)	6 (16)
Antiplatelet	8 (19)	10 (27)
Lipid-lowering agent	15 (36)	13 (35)
Hypoglycemic agent	6 (14)	6 (16)
Baseline bloodwork		
Platelet count, mean (SD)	204 (66)	213 (69)
Glucose, mean (SD), mmol/L	8.6 (3.8)	7.5 (2.5)
Creatinine, mean (SD), mmol/L	95 (65)	107 (114)
Cholesterol, mean (SD), mmol/L	4.4 (0.9)	4.4 (1.4)
ICH characteristics		
Volume, median (IQR), mL	9.9 (0.9-76.9)	15.4 (0.5-122.2)
Deep	36 (86)	30 (81)
Lobar	6 (14)	7 (19)
Intraventricular extension	11 (26)	13 (35)
Clinical examination		
GCS score, median (IQR)	14 (8-15)	15 (6-15)
NIHSS score, median (IQR)	12.5 (0-23)	12.5 (2-27)
Onset to randomization, median (IQR), h	3.1 (2.3)	3.2 (2.1)
Onset to MRI, median (IQR), h	52.2 (17.0-121.4)	51.6 (21.6-107.5)
Baseline vital signs		
SBP, mean (SD), mm Hg	182 (22)	181 (28)
Diastolic BP, mean (SD), mm Hg	97 (18)	92 (14)
Heart rate, mean (SD), bpm	80 (13)	82 (14)

Abbreviations: BP, blood pressure; GCS, Glasgow Coma Scale; MRI, magnetic resonance imaging; NIHSS, National Institutes of Health Stroke Scale; SBP, systolic blood pressure.

SI conversion factors: To convert glucose to mg/dL, divide by 0.0555; cholesterol to mg/dL, divide by 0.0259.

**Table 2.** Baseline Demographic, Intracerebral Hemorrhage (ICH), and Outcome Characteristics of All Randomized Patients

Characteristic	No. (%)		*P* value
	MRI (n = 79)	No MRI (n = 83)	
Demographics			
Age, mean (SD), y	71 (13)	71 (13)	.96
Sex			
Female	36 (46)	39 (47)	.27
Male	43 (54)	44 (53)	.88
Medical history			
Hypertension	56 (71)	61 (74)	.73
Previous ICH	7 (9)	7 (8)	>.99
Previous ischemic stroke	12 (15)	14 (17)	.83
Diabetes	19 (24)	18 (22)	.85
Ischemic heart disease	3 (4)	10 (12)	.08
Smoking (current)	7 (9)	11 (13)	.46
Alcohol use (current)	15 (19)	8 (10)	.12
Medications			
Antihypertensive agent	19 (34)	28 (34)	.23
Anticoagulant	11 (13)	11 (14)	>.99
Antiplatelet	17 (22)	28 (34)	.11
Lipid-lowering agent	31 (39)	29 (35)	.63
Hypoglycemic agent	14 (18)	13 (16)	.83
ICH characteristics			
Volume, median (IQR), mL	11.2 (0.5-122.2)	24.0 (0.3-148.7)	<.001
Deep/brainstem	66 (83)	60 (72)	.09
Lobar	13 (17)	23 (28)	
Intraventricular extension	26 (33)	41 (49)	.04
Clinical examination			
Baseline systolic BP, mean (SD)	182 (25)	182 (25)	.98
GCS score, median (IQR)	15 (6-15)	13 (3-15)	.02
NIHSS score, median (IQR)	12.5 (0-27)	17 (1-29)	.01
Radiographic outcomes			
24-h NCCT scan volume, median (IQR), mL	13.6 (19.2)	34.5 (58.4)	.02
Expansion ≥6 mL^a^	19 (27)	19 (28)	>.99
Expansion ≥33%^a^	18 (25)	21 (31)	.57
Clinical outcomes			
30-d Mortality rate	11 (13)	37 (45)	<.001

Abbreviations: BP, blood pressure; GCS, Glasgow Coma Scale; MRI, magnetic resonance imaging; NCCT, noncontrast computed tomography; NIHSS, National Institutes of Health Stroke Scale.

^a^ Not all patients underwent 24-hour computed tomography, most often due to early death or withdrawal of care.

### BP Treatment

**Figure 2.**
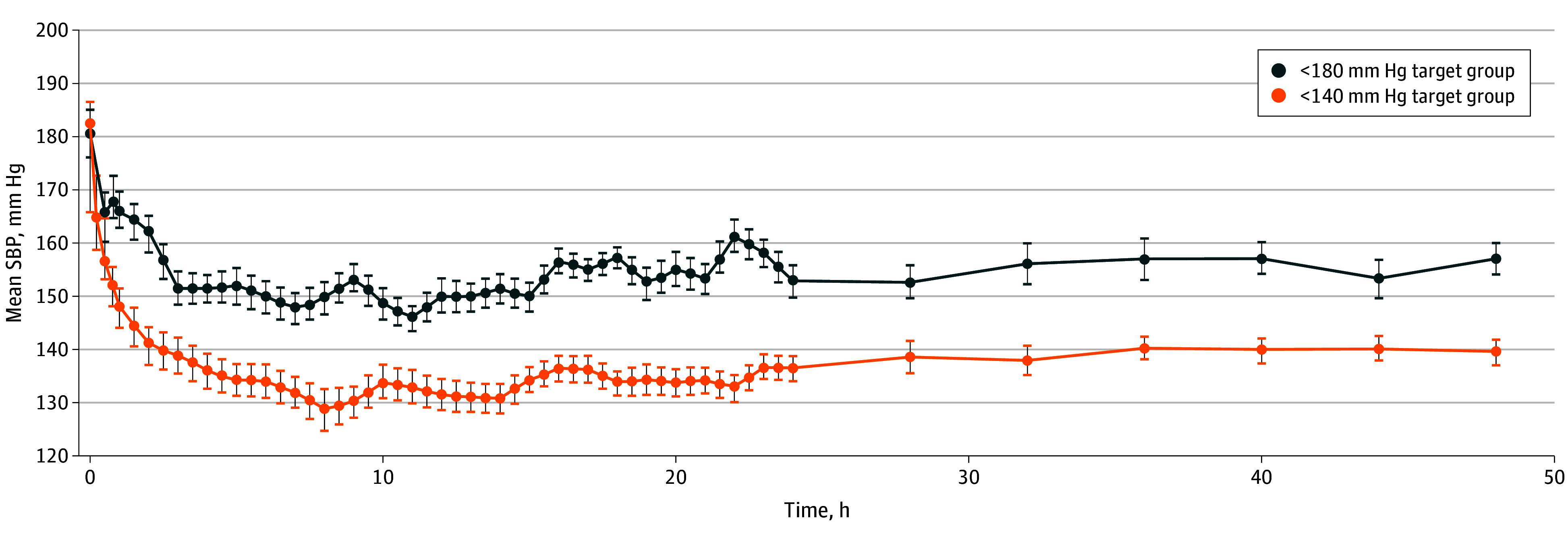
Mean Systolic Blood Pressure (SBP) Over First 48 Hours After Randomization in Both Treatment Groups Blood pressure was monitored at 15-minute intervals for the first hour after randomization (time 0), every 30 minutes from hours 1 to 24, and every 4 hours up to 48 hours. Mean SBP was lower over the 48-hour period after randomization in the less than 140 mm Hg target group (adjusted mean difference for period, 18.9 mm Hg [95% CI, 17.6-20.2]; *P* < .001). Error bars represent standard error of the mean at each time point.

The mean (SD) baseline SBP was 183 (22) mm Hg in the less than 140 mm Hg target group and 181 (28) mm Hg in the less than 180 mm Hg target group. Mean SBP was lower over the 48-hour period after randomization in the less than 140 mm Hg target group (adjusted mean difference for period, 18.9 mm Hg [95% CI, 17.6-20.2]; *P* < .001) (Figure 2).

### Lesion Incidence

The primary outcome of DWI lesion incidence at 48 hours in the less than 140 mm Hg target group was 13 of 42 patients (31%), which was not statistically significantly higher than that in the less than 180 mm Hg group (14/37 [38%]; odds ratio [OR], 0.74 [95% CI, 0.12-4.64]; *P* = .32).

A total of 58 patients underwent MRI within 7 days of symptom onset, including 42 repeat scans. The number of patients with DWI lesions increased by day 7, but the proportions in each BP treatment group were not different (Table 3). Two incident lesions were found in patients undergoing repeat MRI 30 days after onset (1 in each treatment group).

**Table 3.** Primary and Secondary Outcomes Between Treatment Groups

Outcome	Target SBP, No./total No. (%)		OR (95% CI)	*P* value
	<140 mm Hg	<180 mm Hg		
Primary outcome				
DWI lesion incidence, 48 h				
DWI lesion topography	13/42 (31)	14/37 (38)	0.74 (0.12-4.64)	.32
Ipsilateral	7/13 (54)	5/14 (36)		
Contralateral	4/13 (31)	4/14 (33)		
Bilateral	2/13 (15)	5/14 (36)		
Subcortical^a^	6/13 (46)	4/14 (29)		
Cortical	6/13 (46)	6/14 (43)		
Subcortical and cortical	1/13 (8)	4/14 (29)		
Secondary outcomes				
Lesion incidence, 7 d^b^	6/33 (18)	5/25 (21)	0.89 (0.07-10.74)	.50
Cumulative No. of patients with lesions within 7 d^b^	16/49 (33)	18/46 (39)	0.75 (0.14-3.98)	.26
DWI lesion No., median (IQR)^c^	1 (1-10)	1.5 (1-10)		.26
DWI lesion volume, median (range), mL^c^	0.1 (0.01-41.3)	0.3 (0.02-2.03)		.17
Mortality, 30 d	4/42 (10)	7/37 (19)	0.45 (0.12-1.69)	.33

Abbreviations: DWI, diffusion-weighted imaging; MRI, magnetic resonance imaging; OR, odds ratio; SBP, systolic blood pressure.

^a^ Subcortical includes brainstem and cerebellar lesions.

^b^ Incident DWI lesions found in 11 of 58 patients scanned at day 7: 4 of 10 patients with lesions at 24 hours developed additional DWI lesions, 4 of 31 patients without lesions at 48 hours developed new DWI lesions, and 3 of 16 patients without a 48-hour MRI developed DWI lesions.

^c^ Measurements on 48-hour MRI only (n = 26).

### Lesion Number and Volume

The median number of DWI lesions, topographic distribution, and total volume did not differ between groups (Table 3). Most lesions were less than 1 mL. The largest DWI lesion volume was 41.3 mL, involving the internal border zones bilaterally in a patient randomized to the less than 140 mm Hg group (eFigure 1 in Supplement 3).

### Adverse Events

The rate of adverse events for all randomized patients was similar in both treatment groups (37 events in 23/81 patients in the less than 140 mm Hg group and 40 events in 32/81 patients in the less than 180 mm Hg group). Two patients in the less than 140 mm Hg group, but not the DWI analysis, developed ischemic stroke. One patient developed an embolic pattern infarct associated with a large-vessel angiopathy and the other was reported to have evidence of contralateral posterior cerebral artery ischemic infarction secondary to herniation. One patient in the less than 180 mm Hg target group presented with an anticoagulant-associated ICH and developed an embolic pattern ischemic infarct 3 days after admission. In all 162 randomized patients, the early (30-day) mortality rate in the less than 140 mm Hg target group (18/81 [22%]) was not elevated compared with those in the less than 180 mm Hg group (28/81 [35%]; OR, 0.54; *P* = .12).

### Exploratory Analyses of Predictors of DWI Lesion Incidence

Univariable logistic regression indicated that DWI lesions at 48 hours were predicted by total ICH volume, ICH location (lobar vs deep), baseline SBP, and 48-hour weighted mean SBP (eTable in Supplement 3). Higher SBP at baseline and over 48 hours was associated with an increased likelihood of DWI lesions. Inclusion of ICH volume, location, and 48-hour weighted mean SBP in a multivariable logistic regression model indicated that only volume predicted DWI lesion incidence (OR, 1.04 [95% CI, 1.01-1.07]; *P* = .02).

### Meta-Analysis

Systematic review of the literature identified 1 published study using our search terms. The ATACH-2 trial included 171 patients with acute ICH randomized (1:1) to an SBP target of 110 mm Hg to 139 mm Hg (intensive target) or 140 mm Hg to 179 mm Hg (standard treatment), with a DWI scan obtained 1 to 10 days after randomization.^13^ Results of this study and the 79 patients from ICHADAPT-2 (primary end point) were combined in a random-effects meta-analysis. The summary risk ratio when using the ICHADAPT-2 primary outcome of DWI lesion incidence at 48 hours was 0.92 (95% CI, 0.62-1.38) in the combined analysis of 250 patients with acute ICH, without evidence of heterogeneity (*I^2^* = 0%) (eFigure 2 in Supplement 3). Sensitivity analysis using the cumulative incidence of DWI lesions up to 7 days from ICHADAPT-2 yielded a combined sample of 266 patients and a similar risk ratio of 0.90 (95% CI, 0.61-1.31; *I^2^* = 0%).

## Discussion

In this randomized clinical trial, no increase in DWI lesion incidence was seen despite a statistically significant, rapid, and sustained reduction in SBP. DWI lesions were common in both treatment groups, but the majority were small volume. These findings do not support the hypothesis that intensive BP treatment precipitates ischemic injury after ICH.

Neither SBP target nor actual SBP reduction were associated with increased DWI lesion frequency, consistent with the findings of a secondary analysis of patients in the ATACH-2 trial who underwent an MRI within 10 days of randomization.^13^ In ATACH-2, random assignment to a treatment target group (<140 mm Hg or <180 mm Hg) and actual SBP achieved were not associated with increased risk of DWI lesions. Conversely, post hoc analyses of the Minimally Invasive Surgery With Thrombolysis in Intracerebral Hemorrhage Evacuation (MISTIE III) trial indicated a reduction in SBP of 80 mm Hg or greater was associated with a doubling of the odds of DWI lesion incidence, compared with patients with an SBP decrease of less than 20 mm Hg.^14^ Notably, BP reduction and target levels were not randomized in MISTIE III, which introduces susceptibility to residual confounding in the observed associations, similar to prior observational studies. Direct comparison with previous trials is confounded by differences in intervention and population, but the ATACH-2 trial was most similar to the current investigation. Exploratory study-level meta-analysis demonstrates the consistency of the findings in 266 patients randomized across the 2 trials (eFigure 2 in Supplement 3).

Study results and those of previous studies indicate an association between ICH volume and DWI lesions. Patients with DWI lesions in the MISTIE III analysis had a median (IQR) ICH volume of 48 (23) mL, which was nearly 5-fold higher than randomized patients undergoing MRI in ICHADAPT-2 (Table 2). The larger ICH volumes were likely related to the higher DWI lesion incidence rate in MISTIE III (59%) compared with this study (31% and 38% in the less than 140 mm Hg and less than 180 mm Hg target groups, respectively). Similarly, in ATACH-2, ICH volumes greater than 30 mL were associated with increased DWI lesion incidence. The high rate of failure to obtain an MRI in randomized patients with larger ICH volumes in this trial and the relatively smaller ICH volumes studied in most retrospective DWI studies suggests that the incidence of lesions may be currently underestimated due to sampling bias inherent to MRI acquistion.^6,7,8,9,10,11,12,13,14,15,16,17^

A 2023 meta-analysis indicated that DWI lesions were most common in the first 48 hours after ICH, but were also reported beyond this acute period.^26^ The authors hypothesized an interaction between underlying chronic cerebral small-vessel disease and acute precipitants, which may include acute BP variations, cerebral autoregulation impairment related to the ICH, elevated intracranial pressure, and acute hydrocephalus. Although the primary purpose of ICHADAPT-2 was not to determine the mechanism of DWI lesion formation after ICH, the findings are consistent with this hypothesis. The serial imaging results indicated that most DWI lesions develop early after onset, but new lesions continue to develop in some patients even up to 30 days after onset. It is difficult to causally implicate acute BP management over the first 24 hours with the development of these later lesions.

The distribution of DWI lesions after ICH within vascular border zones, often referred to as watershed regions, has been cited as evidence for hypoperfusion-related ischemic injury.^18^ Although not exclusively found in these regions, the largest-volume DWI lesions had bilateral internal border zone distributions (eFigure 1 in Supplement 3). Cerebral perfusion was not measured, but in the first Intracerebral Hemorrhage Acutely Decreasing Arterial Pressure Trial (ICHADAPT), intensive SBP reduction was not associated with changes in cerebral blood flow, measured with computed tomographic perfusion either in the perihematoma region or vulnerable internal and external border zone regions.^27,28^ While a role for acute BP reduction, perhaps acting synergistically with multiple factors, in the mechanism of DWI changes cannot be excluded in all patients, it is likely irrelevant in the majority of cases.

Although BP treatment did not precipitate DWI lesions in ICHADAPT-2, higher baseline SBP was associated with increased risk, consistent with a 2023 meta-analysis.^26^ No studies to date, including the present trial, include the necessary pathophysiological data required to determine if this is a causal association or not. Nonetheless, severe hypertension has been hypothesized to contribute to impairment of cerebral autoregulation in cerebrovascular disease, and this may be relevant in ICH.^12,26^

All DWI lesions were asymptomatic, reflecting both their small volumes and the baseline neurological deficits related to the index ICH. There is some evidence that long-term morbidity and mortality are elevated in patients with ICH with DWI lesions, but the causal association, if any, remains unknown.^26^ Ongoing uncertainties about the true frequency of DWI lesions after ICH, their association with acute interventions, and outcomes may be addressed in the future if routine early MRI scans become part of ongoing ICH registries and practice guidelines.^1,29,30^

### Limitations

This study has limitations. First, the requisite sample size to detect a 22% increase in DWI lesions was not reached and therefore the trial is underpowered to reject the null hypothesis. However, the study-level meta-analysis resulting in a combined sample of 250 patients was consistent with the overall results. Second, there was clear selection bias, such that patients with larger ICH volumes and more severe neurological disability were less likely to undergo DWI (eTable in Supplement 3). This resulted in more than 50% of randomized patients without the primary end point (Figure 1). The effect of acute BP reduction on DWI lesion incidence in the complete ICH population therefore remains unknown. Third, the study did not include assessments for potential embolic sources, notably atherosclerotic stenotic lesions, atrial fibrillation, and angiographic procedures, which have been associated with DWI lesions.^26^ Fourth, the study did not assess for other potential etiological factors, including leukoaraiosis and cerebral microbleeds, which have been associated with DWI lesions.^19^

## Conclusion

DWI lesions were not precipitated or aggravated by intensive BP reduction, supporting the safety of intensive antihypertensive therapy in patients with ICH.

## Notes

Supplement 1. Trial Protocol

Supplement 2. Statistical Analysis Plan

Supplement 3. eTable. Univariable Predictors of DWI Lesions on 48-hour MRI in the Intracerebral Hemorrhage Acutely Decreasing Arterial Pressure 2 Trial (ICHADAPT-2) eFigure 1. Largest diffusion-weighted image (DWI) volume lesions on 48-hour MRI and more typical lesions in the Intensive Treatment Group (Target Systolic Blood Pressure <140 mm Hg; left) and the Conservative Treatment Group (Target Systolic Blood Pressure <180 mm Hg; right) in the Intracerebral Hemorrhage Acutely Decreasing Arterial Pressure 2 Trial eFigure 2. Study-level random-effects meta-analysis of the Intracerebral Hemorrhage Acutely Decreasing Arterial Pressure 2 Trial (ICHADAPT-2) and Antihypertensive Treatment of Acute Cerebral Hemorrhage II (ATACH-2) diffusion-weighted imaged (DWI) lesion secondary analysis

Supplement 4. Data Sharing Statement

## References

[1] Greenberg SM, Ziai WC, Cordonnier C, . 2022 guideline for the management of patients with spontaneous intracerebral hemorrhage: a guideline from the American Heart Association/American Stroke Association. Stroke. 2022;53(7):e282-e361. doi:10.1161/STR.0000000000000407 35579034

[2] Ma L, Hu X, Song L, . The third Intensive Care Bundle with Blood Pressure Reduction in Acute Cerebral Haemorrhage Trial (INTERACT3): an international, stepped wedge cluster randomised controlled trial. Lancet. 2023;402(10395):27-40. doi:10.1016/S0140-6736(23)00806-1 37245517 PMC10401723

[3] Qureshi AI, Palesch YY, Barsan WG, . Intensive blood-pressure lowering in patients with acute cerebral hemorrhage. N Engl J Med. 2016;375(11):1033-1043. doi:10.1056/NEJMoa1603460 27276234 PMC5345109

[4] Anderson CS, Heeley E, Huang Y, . Rapid blood-pressure lowering in patients with acute intracerebral hemorrhage. N Engl J Med. 2013;368(25):2355-2365. doi:10.1056/NEJMoa1214609 23713578

[5] Asdaghi N, Campbell BC, Butcher KS, . DWI reversal is associated with small infarct volume in patients with TIA and minor stroke. AJNR Am J Neuroradiol. 2014;35(4):660-666. doi:10.3174/ajnr.A3733 24335541 PMC7965816

[6] Menon RS, Burgess RE, Wing JJ, . Predictors of highly prevalent brain ischemia in intracerebral hemorrhage. Ann Neurol. 2012;71(2):199-205. doi:10.1002/ana.22668 22367992 PMC3298034

[7] Prabhakaran S, Naidech AM. Ischemic brain injury after intracerebral hemorrhage: a critical review. Stroke. 2012;43(8):2258-2263. doi:10.1161/STROKEAHA.112.655910 22821611

[8] Garg RK, Liebling SM, Maas MB, Nemeth AJ, Russell EJ, Naidech AM. Blood pressure reduction, decreased diffusion on MRI, and outcomes after intracerebral hemorrhage. Stroke. 2012;43(1):67-71. doi:10.1161/STROKEAHA.111.629493 21980211 PMC3246540

[9] Gregoire SM, Charidimou A, Gadapa N, . Acute ischaemic brain lesions in intracerebral haemorrhage: multicentre cross-sectional magnetic resonance imaging study. Brain. 2011;134(Pt 8):2376-2386. doi:10.1093/brain/awr172 21841203

[10] Prabhakaran S, Gupta R, Ouyang B, . Acute brain infarcts after spontaneous intracerebral hemorrhage: a diffusion-weighted imaging study. Stroke. 2010;41(1):89-94. doi:10.1161/STROKEAHA.109.566257 19892994

[11] Kang DW, Han MK, Kim HJ, . New ischemic lesions coexisting with acute intracerebral hemorrhage. Neurology. 2012;79(9):848-855. doi:10.1212/WNL.0b013e3182648a79 22843271

[12] Arsava EM, Kayim-Yildiz O, Oguz KK, Akpinar E, Topcuoglu MA. Elevated admission blood pressure and acute ischemic lesions in spontaneous intracerebral hemorrhage. J Stroke Cerebrovasc Dis. 2013;22(3):250-254. doi:10.1016/j.jstrokecerebrovasdis.2011.08.006 21963218

[13] Shoamanesh A, Cassarly C, Morotti A, . Intensive blood pressure lowering and DWI lesions in intracerebral hemorrhage: exploratory analysis of the ATACH-2 randomized trial. Neurocrit Care. 2022;36(1):71-81. doi:10.1007/s12028-021-01254-9 34292474

[14] Rivera-Lara L, Cho SM, Li Y, . Mechanistic evaluation of diffusion weighted hyperintense lesions after large spontaneous intracerebral hemorrhage: a subgroup analysis of MISTIE III. Neurocrit Care. 2024;40(3):1140-1150. doi:10.1007/s12028-023-01890-3 38040993

[15] Kimberly WT, Gilson A, Rost NS, . Silent ischemic infarcts are associated with hemorrhage burden in cerebral amyloid angiopathy. Neurology. 2009;72(14):1230-1235. doi:10.1212/01.wnl.0000345666.83318.03 19349602 PMC2677484

[16] Auriel E, Gurol ME, Ayres A, . Characteristic distributions of intracerebral hemorrhage-associated diffusion-weighted lesions. Neurology. 2012;79(24):2335-2341. doi:10.1212/WNL.0b013e318278b66f 23197745 PMC3578378

[17] Kidwell CS, Rosand J, Norato G, . Ischemic lesions, blood pressure dysregulation, and poor outcomes in intracerebral hemorrhage. Neurology. 2017;88(8):782-788. doi:10.1212/WNL.0000000000003630 28122903 PMC5344081

[18] Zhang A, Ren M, Deng W, . Ischemia in intracerebral hemorrhage: a comparative study of small-vessel and large-vessel diseases. Ann Clin Transl Neurol. 2022;9(1):79-90. doi:10.1002/acn3.51497 35018741 PMC8791802

[19] Murthy SB, Cho SM, Gupta A, . A pooled analysis of diffusion-weighted imaging lesions in patients with acute intracerebral hemorrhage. JAMA Neurol. 2020;77(11):1390-1397. doi:10.1001/jamaneurol.2020.2349 32687564 PMC7372494

[20] Gioia L, Klahr A, Kate M, . The intracerebral hemorrhage acutely decreasing arterial pressure trial II (ICH ADAPT II) protocol. BMC Neurol. 2017;17(1):100. doi:10.1186/s12883-017-0884-4 28525977 PMC5437568

[21] Kothari RU, Brott T, Broderick JP, . The ABCs of measuring intracerebral hemorrhage volumes. Stroke. 1996;27(8):1304-1305. doi:10.1161/01.STR.27.8.1304 8711791

[22] Fedorov A, Beichel R, Kalpathy-Cramer J, . 3D Slicer as an image computing platform for the Quantitative Imaging Network. Magn Reson Imaging. 2012;30(9):1323-1341. doi:10.1016/j.mri.2012.05.001 22770690 PMC3466397

[23] Harris CR, Millman KJ, van der Walt SJ, . Array programming with NumPy. Nature. 2020;585(7825):357-362. doi:10.1038/s41586-020-2649-2 32939066 PMC7759461

[24] Virtanen P, Gommers R, Oliphant TE, . SciPy 1.0: fundamental algorithms for scientific computing in Python. Nat Methods. 2020;17(3):261-272. doi:10.1038/s41592-019-0686-2 32015543 PMC7056644

[25] Seabold S, Perktold J. Statsmodels: econometric and statistical modeling with Python. Paper presented at: Proceedings of the 9th Python in Science Conference; June 28, 2010; Austin, TX. Accessed March 10, 2025. doi:10.25080/Majora-92bf1922-011

[26] Posener S, Hmeydia G, Benzakoun J, Oppenheim C, Baron JC, Turc G. Remote diffusion-weighted imaging lesions and intracerebral hemorrhage: a systematic review and meta-analysis. Stroke. 2023;54(4):e133-e137. doi:10.1161/STROKEAHA.122.040689 36866676

[27] Butcher KS, Jeerakathil T, Hill M, . The intracerebral hemorrhage acutely decreasing arterial pressure trial. Stroke. 2013;44(3):620-626. doi:10.1161/STROKEAHA.111.000188 23391776

[28] Gould B, McCourt R, Gioia LC, . Acute blood pressure reduction in patients with intracerebral hemorrhage does not result in borderzone region hypoperfusion. Stroke. 2014;45(10):2894-2899. doi:10.1161/STROKEAHA.114.005614 25147326

[29] Hemphill JC III, Adeoye OM, Alexander DN, . Clinical performance measures for adults hospitalized with intracerebral hemorrhage: performance measures for healthcare professionals from the American Heart Association/American Stroke Association. Stroke. 2018;49(7):e243-e261. doi:10.1161/STR.0000000000000171 29786566

[30] Li Q, Yakhkind A, Alexandrov AW, . Code ICH: a call to action. Stroke. 2024;55(2):494-505. doi:10.1161/STROKEAHA.123.043033 38099439

