# Supplementary material for: Acute Blood Pressure Lowering and Risk of Ischemic Lesions on MRI After Intracerebral Hemorrhage
Source: JAMA Neurol. 2025 Apr 21;82(6):543–50. doi: 10.1001/jamaneurol.2025.0586 (PMC12012699; doi:10.1001/jamaneurol.2025.0586)
Supplement: Supplement 1. — Trial Protocol [file jamaneurol-e250586-s001.pdf]

The Intracerebral Hemorrhage Acutely Decreasing Arterial Pressure Trial II

ICH-ADAPT II 17 Feb 2020 Version 3.3

Sponsor: Investigator Initiated Trial

Principal Investigator: Professor Ken Butcher

Prince of Wales Clinical School
Level 1, South Wing, Edmund Blacket Building
Prince of Wales Hospital
Randwick NSW 2031

Phone: 02 9382 7914
Fax: 02 9382 8885
Email: ken.butcher@unsw.edu.au

SIGNATURE PAGE

The signature below constitutes the approval of this protocol and the attachments, and provides the necessary assurances that this trial will be conduct according to all stipulations of the protocol, including all statements regarding confidentiality and according to local legal and ethical requirements and applicable Australian regulations and International Consensus on Harmonisation guidelines.

Qualified Investigator/Principal Investigator

Name: ________________________________

Signed: ________________________________ Date: ___________________(dd/mmm/yyyy)

Site Name: ___________________________________

Address: ___________________________________

____

____

____

TABLE OF CONTENTS

List Of Abbreviations 5

Protocol Summary 7

1.0 Background And Rationale 9

1.1 Pathophysiology. 9

1.2 Ich Volume And Expansion. 9

1.3 Blood Pressure And Ich. 9

1.4 Rationale For Acute Blood Pressure Reduction. 10

1.5 Rationale For Conservative Blood Pressure Management. 10

1.6 Current Practice Guidelines. 11

1.7 Completed And Ongoing Acute Blood Pressure Treatment Trials In Ich Patients. 11

1.7.1 Ich Adapt: 11

1.7.2 Interact: 12

1.7.3 Atach: 13

1.8 New Evidence For Delayed Ischemia In Ich. 13

1.9 Knowledge Gaps. 14

2.0 Objectives Of Study 15

2.1 Endpoints 15

2.1.1 Primary Endpoint: 15

2.1.2 Secondary Endpoints: 15

2.2 Overall Study Aim: 15

2.3 Hypothesis: 16

3.0 Study Design 16

4.0 Enrolment Criteria 16

4.1 Inclusion Criteria 16

4.2 Exclusion Criteria 16

4.3 Inclusion/Exclusion Criteria Considerations 17

5.0 Randomization 17

6.0 Treatment: Bp Management Protocols 17

6.1 "Aggressive" Bp Target (<140mmhg) Treatment Group 17

6.2 "Conservative" Bp Target (<180mmhg) Treatment Group ... 17
6.3 Treatment Considerations ... 18
6.4 Standard Stroke Care ... 18
6.5 Oral Anticoagulant Associated Sub-Group ... 18
6.6 Surgical Management ... 19
7.0 Study Procedures And Evaluations ... 19
7.1 Clinical Screening/Baseline ... 19
7.1.1 Consent Procedures: ... 20
7.2 Study Imaging Procedures ... 20
7.2.1 Baseline: ... 20
7.2.2 24-Hour Ct Scan: ... 20
7.2.3 48-Hour Mri Scan (Primary Endpoint): ... 20
7.2.4 Day 7 Mri Scan (Secondary Endpoint): ... 21
7.2.5 Day 30 Mri Scan (Secondary Endpoint): ... 21
7.3 Clinical Follow-Up Assessments ... 21
7.3.1 In Hospital: ... 21
7.3.2 Day 30 Assessments: ... 22
7.3.3 Day 90 Assessments: ... 22
8.0 Retrospective Study At Powh ... 22
9.0 Statistical Analysis And Power Calculation ... 22
10.0 Central Imaging And Adjudication Of Analysis ... 23
11.0 Trial Management ... 24
11.1 Coordinating Centre ... 24
12.0 Data Safety Monitoring Board ... 24
13.0 Adverse Event Reporting ... 24
14.0 Additional Information ... 24
14.1 Feasibility ... 24
14.2 Significance And Clinical Impact ... 25
15.0 Data Handling And Record Keeping ... 25
16.0 Ethical Considerations ... 26
References ... 27

LIST OF ABBREVIATIONS

AAICH	Anticoagulant-associated intracerebral hemorrhage
ADC	Apparent diffusion coefficient
AE	Adverse event
BBB	Blood brain barrier
BP	Blood pressure
CBF	Cerebral blood flow
CBV	Cerebral blood volume
CRF	Case report form
CT	Computed tomography
CTA	Computed tomography angiography
DSMB	Data safety monitoring board
DWI	Diffusion weighted imaging
eCRF	Electronic case report form
eGFR	Estimated glomerular filtration rate
EPI	Echoplanar imaging
EuroQOL	European Quality of Life
EVD	Extra-ventricular drain
FFP	Fresh frozen plasma
FLAIR	Fluid-Attenuated inverse recovery
GCS	Glasgow Coma Scale
GRE	Gradient recalled echo
HR	Heart rate
HT	Hemorrhagic transformation
ICH	Intracerebral hemorrhage
ICP	Intracranial pressure

IV	Intravenous
IVH	Intraventricular hemorrhage
MAP	Mean arterial pressure
MoCA	Montreal Cognitive Assessment
MRA	Magnetic resonance angiography
MRI	Magnetic resonance imaging
mRS	Modified rankin scale
MTT	Mean transit time
NIHSS	National Institutes of Health Stroke Scale
NCCT	Non-contrast computed tomography
NPO	Nil per os (nothing by mouth)
PCC	Prothrombin complex concentrate
PWI	Perfusion weighted imaging
rCBF	Relative cerebral blood flow
SAE	Serious adverse event
SBP	Systolic blood pressure
SWI	Susceptibility weighted imaging
TE	Repetition time
TR	Spin echo time

PROTOCOL SUMMARY

Title: ICH ADAPT II

The **I**ntra**c**erebral **H**emorrhage **A**cutely **D**ecreasing Blood **P**ressure **T**rial II

ICH ADAPT II is a multi-centre randomized open-label, blinded-endpoint trial for patients with acute ICH, who will be randomized 1:1 to the Aggressive BP target (<140 mmHg) or the Conservative BP target (<180 mmHg). The summary of procedures is outlined in Appendix A.

Overall Study Aim: The primary study aim is to assess the rate of ischemic lesion development in patients randomized to two different BP treatment strategies.

Primary Endpoint: DWI lesion frequency on the first MRI (48 hours)

Secondary Endpoints: The major secondary endpoint is **cumulative DWI lesion rate frequency within 30 days.** Other secondary endpoints include **absolute hematoma growth,** prediction of **DWI lesion incidence, 30-day mortality rates, day 90 functional outcome,** and **cognitive status.**

Population: 270 participants will be enrolled over 2 years. Males and females, **>18 years** of age, with diagnosis of acute **ICH**, confirmed by routine head CT scan**, within 6 hours** of symptom onset. All patients must have two systolic BP measurements ≥ 140 mmHg recorded >2 mins apart and **hematoma volume <90 mL** (ABC/2) to qualify for enrolment.

Phase: II

Number of Sites: This is a multi-centre investigator-initiated study, coordinated by Professor Butcher. Prince of Wales Hospital is one of the participating centres. The other participating centres are as follows: University of Alberta Hospital, Edmonton, AB, Canada Hamilton Health Sciences Centre, Hamilton ON, Canada, Ottawa Hospital, Ottawa, ON, Canada

Protocol Therapy: Patients will be randomized 1:1 to aggressive treatment of BP to <140 mmHg versus conservative treatment of BP to <180 mmHg. Patients randomized to the <140 mmHg group will immediately receive a 10mg IV bolus of labetalol, administered over 1 minute. Patients randomized to the <180 mmHg will only be administered parenteral antihypertensive therapy if systolic BP is ≥180 mmHg.

Study Duration: It is estimated that recruitment will take place over 2 years in the Prince of Wales Hospital and the other three participating centres. **Subject Participation Duration**: All patients will be assessed clinically at Day 30 and Day 90.

ICHADAPT II Schedule of Events

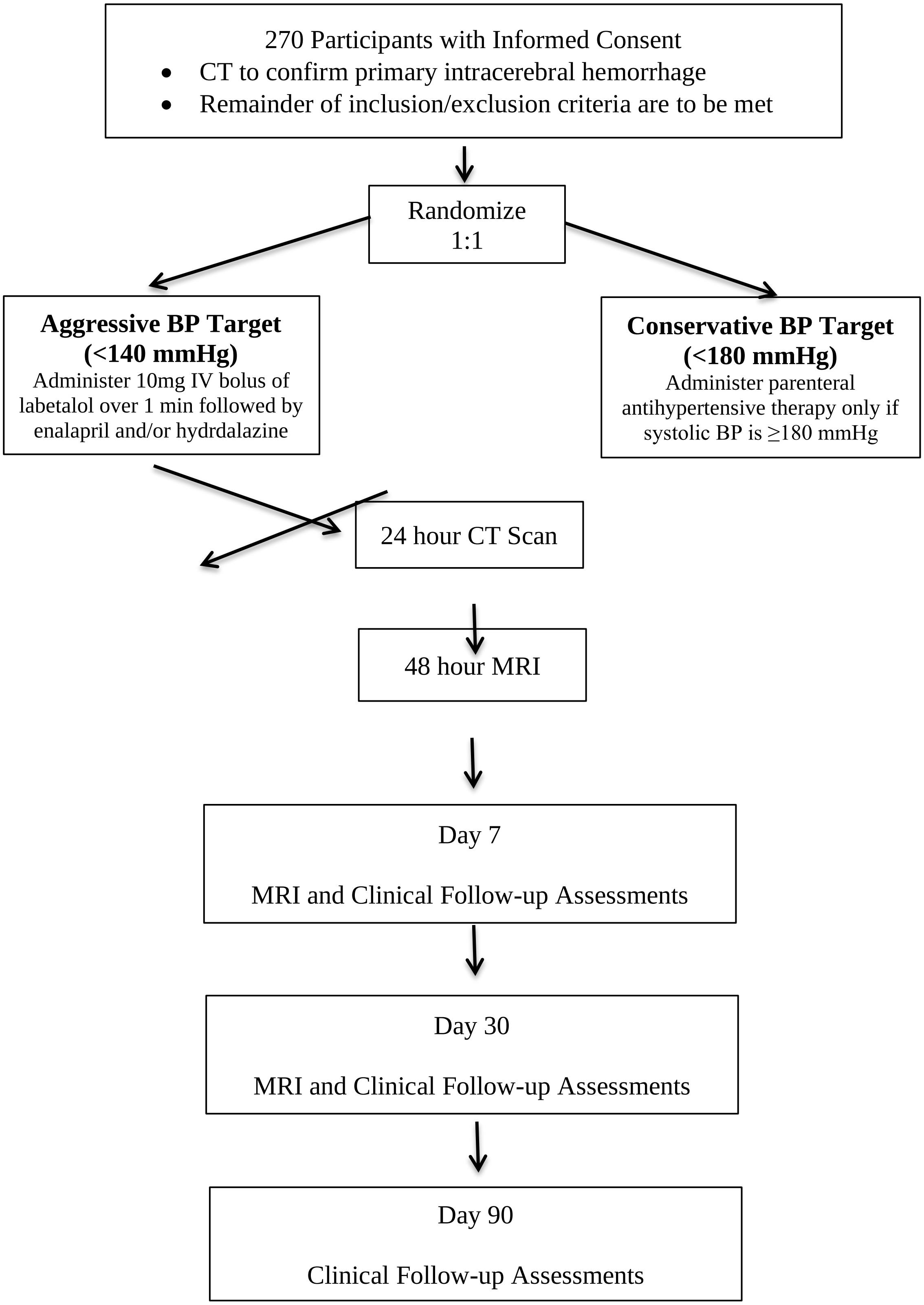

1.0 BACKGROUND AND RATIONALE

Primary intracerebral hemorrhage (ICH) refers to spontaneous, non-traumatic bleeding from intraparenchymal blood vessels, pathologically altered by long-standing hypertension or cerebral amyloid angiopathy.[1] The worldwide annual incidence of ICH is 13-30 per 100 000, or 2-3 million people.[1-7] In Canada, approximately 5440 new cases of ICH occur each year.[8] Bleeding from vascular malformations or neoplasia—classified as secondary intracerebral hemorrhage—represents less than 20% of all ICH.[9] Therapeutic anticoagulants are also associated with an increased rate of ICH, although classification can be problematic, as most patients also have other risk factors.[10, 11] Hemorrhages of this nature are therefore described as anticoagulant-associated ICH (AAICH). Early mortality rates are significantly higher in ICH, relative to ischemic stroke. Approximately 30-50% of patients die within 30 days of onset, most in the initial 48 h, and the majority of survivors are left with long-term disability.[12-16] Despite the socio-economic burden, acute treatment options are limited. The role of surgery is controversial, but current evidence indicates hematoma evacuation confers no benefit over medical management.[17] No medical therapies have been proven effective and several have been shown to be unhelpful including steroids, osmotic diuretics and hemodilution.[18-21]

1.1 Pathophysiology.

ICH can be classified by neuroanatomical location, as deep or lobar. Deep ICH result from rupture of arteries/arterioles affected by fibrohyalinosis, due to longstanding hypertension, and occur most commonly in the putamen, thalamus, caudate, cerebellum and pons.[3, 22] Lobar ICH may be related to fibrohyalinosis and/or amyloid angiopathy.[23] Upon vessel rupture, blood extravasates into brain parenchyma, introducing compressive and shear forces that result in the primary cerebral injury and rupture of additional vessels.[24]

1.2 ICH Volume and Expansion.

Initial ICH volume is highly predictive of early mortality. ICH volumes >50 ml are associated with 30-day mortality rates of 90%.[25, 26] Hematoma volume is not static in all patients (*Figure 1*). Many patients continue to bleed during the first few hours resulting in continued ICH enlargement and rapid clinical deterioration.[27, 28] Retrospective studies indicate ICH expansion continues after symptom onset in 20-36% of patients.[29, 30] The vast majority of expansion appears to occur within hours of onset. In a prospective study, significant ICH expansion (>33% volume 1 h after baseline scan) was observed in 26% of patients initially imaged within 3 h of onset.[31] ICH expansion is a strong independent predictor of neurological deterioration, death and impaired functional recovery.[32-35] Prevention of expansion is the most logical acute ICH treatment target.

1.3 Blood Pressure and ICH.

Hypertension is the single most important modifiable risk factor for ICH. Elevated BP increases the risk of ICH by 2-6 fold.[6, 11, 36-38] Antihypertensive therapy reduces the risk of ICH by up to

50%.[39-46] Stroke physicians are increasingly aggressive in treating chronic hypertension in ICH survivors for long-term secondary stroke prevention. The most pressing remaining controversy is management of BP in the acute phase of ICH. Hypertension (SBP>140 mmHg) is extremely common in the acute stages of ICH, occurring in up to 90% of patients.[47-49] Pressures are highest initially and decline spontaneously in the first 24 h after onset.[47, 48] Outcome has been correlated with acute BP in ICH.[50-61] It has been clearly demonstrated that patients with higher BP at presentation have elevated early mortality rates.[50, 58] Outcome and acute BP appeared to be linearly related, with minimal or no increases in morbidity/mortality associated with lower pressures.[50, 62] Causality is impossible to determine from these observational studies. Available data support the hypothesis that higher BP contributes to worse outcome, but it is also possible that acute hypertension is a homeostatic response to elevated intracranial pressure (ICP).[63] This dilemma has hampered development of a rational approach to acute BP management. Optimally designed randomized treatment trials are urgently required to determine the effects of BP reduction on ischemic injury as well as outcome.

1.4 Rationale for Acute Blood Pressure Reduction.

An intuitive and biologically plausible potential benefit of BP reduction is attenuation of ICH growth. Indeed, multivariate analyses indicate a strong correlation between elevated systolic BP and subsequent ICH expansion.[33, 35] Furthermore, acute BP reduction has been associated with a decreased incidence of expansion in retrospective analyses.[64, 65] In a Japanese centre, where aggressive BP reduction (*target systolic BP ≤150 mmHg*) is part of routine care, expansion was seen in only 17% of patients.[65] Another centre reported that a target BP of ≤150 mmHg was associated with expansion in 9% of cases, compared with 30% in those with higher targets.[64] Nonetheless, these non-randomized studies are hypothesis generating only. The best evidence for attenuation of ICH expansion resulting from acute BP reduction comes from the INTensive Reduction of blood pressure in Acute Cerebral haemorrhage Trial (INTERACT).[66] Acute ICH patients (n=346 with evaluable CT scans at baseline and 24 hours) were randomized to <140 mmHg versus <180 mmHg systolic BP target groups within 6 hours of symptom onset. There was a trend to lower rates of ICH expansion in the more intensively treated group (15% versus 23% in <180 mmHg target group). A *post-hoc* sub-group analysis revealed that the greatest effects were seen in patients treated within 3 hours of onset (12% versus 27% growth rate in the <140 mmHg and <180 mmHg target groups respectively).[66] A small (n=60) non-randomized trial (ATACH—Acute Treatment of Acute Cerebral Hemorrhage Trial) assessed the effect of lowering systolic BP to three target tiers ranging from 110 to 200 mmHg, using nicardipine infusions.[67] Although there were no significant effects on ICH expansion, this was trial was designed only as a feasibility study. Follow-up trials (INTERACT II[68] and ATACH II[69]) aimed at determining the effect of BP reduction on ICH expansion are underway.

1.5 Rationale for Conservative Blood Pressure Management.

Physicians have been reluctant to aggressively reduce BP in the acute phase of ICH. This is predicated on the belief that there might be a zone of ischemia surrounding the acute hematoma.[70] Although there is no convincing evidence of perihematoma injury resulting from ischemia, it has been postulated that this tissue may be more vulnerable to decreased BP,

which could produce or exacerbate ischemic conditions, adversely affecting outcome.[71] The localized mass effect of a hematoma may also result in an asymmetric rise in ICP and subsequently herniation.[72] It has been suggested that these patients are at risk of cerebral blood flow (CBF) compromise secondary to any reduction in BP.[28] Finally, it has been hypothesized that cerebral autoregulation in acute ICH is disrupted, making CBF more passively dependent on BP. It should be noted that while studies like INTERACT and ATACH have not been associated with excess clinical adverse events, these trials do not include physiological markers of blood flow or ischemic injury. ***This is important, as adverse effects may not be obvious in patients already suffering from severe neurological deficits secondary to the initial ICH.***

1.6 Current Practice Guidelines.

In the absence of evidence favouring aggressive or conservative treatment strategies, physicians have been forced to make empirical decisions based on competing rationales for BP management. Practice guidelines reflect this uncertainty.[73-75] Guidelines recognize excessively high BP is likely harmful but also acknowledge the possibility of cerebral ischemia following aggressive reduction, resulting in conservative recommendations. The American Stroke Association recommends intravenous (IV) antihypertensive therapy only when systolic BP is ≥180 mmHg, diastolic BP is ≥110 mmHg or MAP is ≥130 mmHg.[73] In 2010, guidelines were amended to indicate that BP reduction to <160 mmHg could be considered in patients where elevated ICP was not suspected, and that BP reduction to 140 mmHg is 'probably safe'.[76] *Guideline statements also recognize the lack of evidence for any BP target and indicate further research is a priority.*

We conducted a retrospective analysis of BP management in the 142 ICH patients admitted to the University of Alberta in 2005-06.[77] Emergency physicians assigned a BP target in 26.6% of cases. After assessment by the admitting service, 76% of patients were assigned a BP target. *Targets were always based on systolic BP and ranged widely from 110 to 220 mmHg. The assigned target BP was achieved in only 39% of cases at 1 h and 56% at 12 h*. Thus, practice is highly variable, current treatment guideline adherence rates are low, and successful implementation is even less common. There is a clear need for more precise, evidence-based BP treatment guidelines.

1.7 Completed and Ongoing Acute Blood Pressure Treatment Trials in ICH Patients.

1.7.1 ICH ADAPT:

Intracerebral Hemorrhage Acutely Decreasing Arterial Pressure Trial. In order to address concerns related to hypoperfusion following acute BP reduction, we designed and conducted a multicentre randomized controlled trial of aggressive versus conservative BP management strategies, in which the primary endpoint was CBF, measured with CT perfusion imaging. The Intracerebral Hemorrhage Acutely Decreasing Arterial Pressure Trial (ICH ADAPT; clintrials.gov registration number NCT00963976; funding: Alberta Innovates Health Solutions (G513000128), Heart and Stroke Foundation

of Canada (G220170180)) randomized patients to target systolic BP of <150 mmHg or <180 mmHg.[78] Patients were randomized to a target systolic BP of <150 mmHg (n=39) or <180 mmHg (n=36). Mean systolic BP two hours after randomization was significantly lower in the <150 mmHg target group (140±19 vs 162±12 mmHg, p<0.001, *Figure 2*). Perihematoma CBF (38.7±11.9 ml/100g/min) was lower than in contralateral homologous regions (44.1±11.1 ml/100g/min, p<0.001) in all patients. The primary endpoint of perihematoma rCBF in the <150 mmHg target group (0.86±0.12) was not significantly lower than that in the <180 mmHg group (0.89±0.09, p=0.19; absolute difference 0.03 95% CI -0.018–0.078). Ipsilateral hemispheric rCBF was however mildly lower in patients randomized to the <150 mmHg target group (0.95±0.05), relative to those in the <180 mmHg target group (0.99±0.05, p=0.0013). The effects of BP treatment did not vary with hematoma location, volume or time to initiation of antihypertensive therapy. A sub-study of ICH ADAPT suggests that cerebral autoregulation in acute ICH may not be as impaired as once believed. In 20 patients, measurements of CBF were obtained prior to and after treatment with antihypertensive agents. There was no relationship between the magnitude of acute BP reduction and changes in perihematoma rCBF (β=0.001 [-0.002, 0.003], P=0.63; *Figure 3*). *In summary, ICH ADAPT I demonstrated no evidence that rapid BP lowering in acute ICH precipitates ischemia within 2 hours of treatment. The significance of the modest decrease in hemispheric rCBF is unknown, but may potentially be related to delayed ischemic injury in regions topographically remote from the hematoma.*

1.7.2 INTERACT:

INTensive Reduction of blood pressure in Acute Cerebral haemorrhage Trial. The best evidence for attenuation of ICH expansion resulting from acute BP reduction comes from the vanguard phase of INTERACT.[66] Acute ICH patients (n=346 with evaluable CT scans at baseline and 24 hours) were randomized to <140 mmHg versus <180 mmHg SBP target groups within 6 hours of symptom onset. There was a trend to lower rates of ICH expansion in the more intensively treated group (15% versus 23% in <180 mmHg target group). A post-hoc analysis revealed that the greatest effects were seen in patients treated within 3 hours of onset (12% versus 27% growth rate in the <140 mmHg and <180 mmHg target groups respectively).[66] In INTERACT II, 2839 ICH patients were randomized within 6 h of onset to a SBP target of <140 mmHg or <180 mmHg, using a variety of locally available antihypertensive agents.[68, 79] The primary endpoint was death or disability (mRS score 3-6). The rate of death/disability was similar in the <140 mmHg target group (52%) and the <180 mmHg target group (55.3%; OR with intensive treatment, 0.87; 95% CI, 0.75 to 1.01; P=0.06). Intensive BP reduction appeared to be safe, as adverse event and mortality rates were virtually identical in the two treatment groups. The SBP separation between groups was significant (14 mmHg), but only 1/3 of patients in the intensive group achieved the target of <140 mmHg 1 h after randomization.

1.7.3 ATACH:

***Acute Treatment of Acute Cerebral Hemorrhage Trial*.** This open label multicentre trial (n=60) assessed the feasibility and safety of lowering SBP to three target tiers ranging from 110 to 200 mmHg, using IV nicardipine infusions.[67] Nicardipine is an IV dihyrdopyrine calcium channel blocker indicated for BP reduction in acute stroke patients. The ATACH trial demonstrated that SBP can be maintained below treatment threshold within a narrow range using the nicardipine treatment protocol *(Figure 4).* A large SBP decrease was observed at 1 h and 2-24 h in all three treatment tiers. ATACH II is an ongoing randomized controlled trial aimed at determining the effect of BP reduction with IV nicardipine on ICH expansion and clinical outcome (ClinicalTrials.gov registration number NCT01176565).[69] The primary hypothesis being tested is that intensive SBP reduction with IV nicardipine will reduce death or disability (modified Rankin Score 4-6) assessed at 90 days by 10%, relative to the standard of care (target SBP <180 mmHg).

1.8 New Evidence for Delayed Ischemia in ICH.

In contrast to our own MRI and CT perfusion studies, a number of MRI studies published in the last two years have suggested that delayed cerebral ischemia is common within the first week after ICH.[80-86] Diffusion-weighted imaging (DWI) is a specialized MRI sequence that is highly sensitive for ischemic injury.[87, 88] DWI is based on visualization of cerebral water diffusion. In regions that are bio-energetically compromised, due to ischemia, water diffusion slows. This is referred to as diffusion restriction and occurs in regions where ATPase dependent sodium-potassium pumps have failed, resulting in an influx of sodium and water into neurons and glial cells. This is associated with cell swelling and compartmentalization of water, which slows or restricts its diffusion. Tissue that is bio-energetically compromised develops high signal intensity on DWI images within minutes of the onset of ischemia. A DWI study performed by the PI in acute ICH patients indicated that diffusion within the perihematoma region is elevated, suggesting the edema is vasogenic, rather than cytotoxic, i.e. ischemic in origin.[89] Although small areas of perihematoma diffusion restriction have been reported, these have been attributed to non-ischemic, i.e. mechanical or inflammatory, bio-energetic compromise.[90]

More recently, several investigators have reported that discrete areas of diffusion restriction, consistent with cerebral ischemia are found in areas topographically remote to the hematoma, when MRI is performed within one week of symptom onset in 14 - 41 % of patients (Table 1).[80-83, 85, 86] Lesions occur both ipsilateral and contralateral to the hematoma and are often multiple (*Figure 5*). ***Although generally small, DWI lesions have been shown to be clinically significant.*** Two longitudinal studies reported that probability of death/dependence at one year was increased between five and six-fold in ICH patients with DWI lesions.[91, 92] ***Most importantly from the perspective of acute management is the observation that these ischemic lesions are seen more frequently in patients with lower baseline BP and/or following aggressive BP reduction.***[80, 91, 93] However, these were all retrospective observations made in patients where BP treatment decisions were made in a non-randomized manner. Other investigators have reported that DWI lesions are not associated with BP reduction and another indicated these

lesions were actually more frequent in patients with higher presenting BP.[94] The relationship between BP treatment and DWI lesion development is therefore unclear. This question can only be definitively addressed using a randomized study design.

It has also been reported that DWI lesions are often present within watershed vascular territories, suggesting a hemodynamic mechanism. These investigators hypothesize that lower cerebral perfusion pressure following BP reduction results in ischemia within the most vulnerable tissue at the internal and external border-zones between major cerebral arteries. We have not observed borderzone CBF decreases in ICH ADAPT I (*Figure 6*), but it is possible that flow in these regions falls subacutely, leading to DWI lesion development.[80] DWI lesions have also been postulated to result from a thrombotic process, potentially related to the underlying vasculopathy that caused the initial ICH, i.e. lipohyalinosis.[80, 94] Other potential mediators of ischemic injury following ICH include increased platelet activity, withdrawal of antithrombotic therapy, endothelial dysfunction, inflammation and hypercoagulability, but no biomarker studies have been completed to date.[80]

<u>1.9 Knowledge Gaps.</u>

At this point, rapid BP lowering appears to be safe. There are no obvious clinical deficits associated with rapid BP reduction and we have shown that CBF remains stable. The hypothesis that BP treatment improves clinical outcomes via a reduction in ICH expansion remains unproven however. The ATACH II trial is aimed at proving this hypothesis. Unlike, INTERACT II, ATACH II includes only patients who can be treated within 4.5 h (versus 6 h), when ICH expansion is most likely to occur. In addition, IV nicardipine infusions have been shown to be highly effective in lowering SBP in acute ICH patients *(Figure 5)*. The actual BP reduction achieved in INTERACT II was relatively modest. This is unlikely to occur in ATACH II. This trial therefore appears to be more likely to result in a positive outcome with respect to ICH expansion. Although INTERACT II did not reveal an excess of clinical adverse events, it did not include physiological markers of blood flow or ischemic injury. ***This is important, as the clinical effects of new ischemic lesions may not be obvious in patients already suffering from severe neurological deficits secondary to the initial ICH.***

Supported by a planning grant from the Canadian Stroke Network, members of the ICH ADAPT Steering Committee met in Ottawa in 2011 to plan BP and ICH studies in Canada. Demonstration of treatment efficacy and elucidation of the relationship between ischemic lesion formation and BP reduction were identified as research priorities. After publication of the INETRACT II study, we conducted an online survey of Canadian academic stroke community, which indicated that the majority of clinicians believe BP management after ICH remains an area of equipoise even after publication of the INTERACT II results. Of 40 respondents, 93% believed further BP treatment trials in ICH were warranted. In the absence of randomization, respondents indicated they were currently treating to SBP targets of <140 mmHg (50%), <150 mmHg (17%) or <180 mmHg (33%), reflecting persisting uncertainty.

2.0 OBJECTIVES OF STUDY

2.1 Endpoints

2.1.1 Primary Endpoint:

The primary endpoint is DWI lesion frequency on the first MRI (48 hours). *The trial has been designed to test the hypothesis that proportional rates of DWI lesion development will be higher in patients randomized to a systolic BP target <140 mmHg*. The 48 hour MRI has been chosen as the primary endpoint, as previous studies indicate this is the most likely time to detect DWI lesions. Although new lesions will develop in some patients at the time of the second and third MRI scans, these are secondary endpoints. This is a pragmatic decision, as the high mortality rates in ICH will lead to progressively fewer patients being scanned at each time point in a non-random fashion. It is recognized that this will also affect the primary endpoint, but this is an unavoidable consequence of studying physiological endpoints in a disease with high early mortality rates.

2.1.2 Secondary Endpoints:

1. *Cumulative DWI lesion rate frequency within 30 days.* Hypothesis: the frequency of DWI lesions will be higher in the <140 mmHg target BP group.
2. *Absolute hematoma growth.* Hypothesis: mean ICH growth on the 24 h NCCT scan will be significantly lower in the <140 mmHg target BP group compared to the <180 mmHg group.
3. *Prediction of DWI Lesion Incidence.*
   Hypothesis: DWI lesion incidence will be associated with larger hyper-intense white matter volumes measured on FLAIR MRI scans.
   Hypothesis: DWI lesion incidence will be associated with elevated eNOS- platelet counts.
4. *30-day mortality rates.* Hypothesis: 30-day mortality rate will be higher in patients with DWI lesions. It is further hypothesized that this effect will be independent of BP treatment group.
5. *Day 90 Functional Outcome.* Hypothesis: median mRS will be higher in patients with DWI lesions. It is further hypothesized that this effect will be independent of BP treatment group.
6. *Cognitive Status.* Hypothesis: median MoCA scores will be lower in patients with DWI lesions.

2.2 Overall Study Aim:

The primary study aim is to assess the rate of ischemic lesion development in patients randomized to two different BP treatment strategies

2.3 Hypothesis:
Our overall a priori hypothesis is that aggressive BP reduction will not be associated with ischemic injury after ICH.

2.0 STUDY DESIGN

The Intracerebral Hemorrhage Acutely Decreasing Arterial Pressure Trial II (ICH ADAPT II) is a multi-centre randomized open-label, blinded-endpoint trial of two different BP management strategies. This study is being conducted in the Emergency Departments and Stroke Units of Canadian academic and non-academic centres, and in the Prince of Wales Hospital.

4.0 ENROLMENT CRITERIA

4.1 Inclusion Criteria

1. Age ≥ 18 years
2. Two BP measurements ≥ 140 mmHg recorded >2 minutes apart
3. Acute primary ICH with consistent history and CT scan confirmation
4. Hematoma volume on CT must be <90 mL, as estimated using the ABC/2 method
5. Onset ≤6 hours prior to randomization
6. GCS ≥5 prior to randomization

4.2 Exclusion Criteria

1. Known definite CONTRAINDICATION to the BP reduction protocol (i.e. severe arterial stenosis, Moya Moya disease or Takayasu’s arteritis or high-grade stenotic valvular heart disease)
2. Known definite INDICATION for BP reduction (i.e. hypertensive encephalothopy or aortic dissection)
3. Known definite contraindication to MRI (i.e. cardiac pacemaker)
4. Definite evidence that the ICH is secondary to a structural brain abnormality (i.e. AVM, aneurysm, tumor, trauma or hemorrhagic transformation of ischemic infarct).
5. Previous ischemic stroke within 90 days of current event (NB: Prior ICH is not a contraindication)
6. Known history of intracranial neoplastic or vascular lesion
7. Subdural, subarachnoid or epidural hemorrhage
8. Planned resection of hematoma
9. Pre-existing disability and dependence (defined as pre-morbid Modified Rankin Scale Score ≥3)
10. Life expectancy <6 months due to pre-morbid conditions/terminal illness
11. Pervious participation in this trial
12. Current participation in another interventional trial

4.3 Inclusion/Exclusion Criteria Considerations

- All primary ICH patients, irrespective of cerebral location (lobar/subcortical or brainstem) or ICH volume, as well as anticoagulant-associated hemorrhages will be eligible.
- Patients taking warfarin at the time of the ICH will be eligible, but the INR value must be <1.5 prior to randomization
- Placement of an extraventricular drain will be recorded, but is not an exclusion criterion.
- In cases where the treating physician feels there is no hope for survival, or meaningful recovery and palliative care discussions have begun, patients will be excluded.

5.0 RANDOMIZATION

Treatment allocation will be determined using a minimal sufficient balance randomization scheme. This is a unique aspect of the trial, which is designed to ensure balanced treatment groups with respect to enrolling site, age, GCS, baseline ICH volume (which will be estimated using the validated ABC/2 method[95]), anticoagulant use and presence or absence of intraventricular hemorrhage. These prognostic variables reflect the main independent predictors of mortality, as per Hemphill's ICH score.[96-98] A centralized web based randomization process (developed on Adobe Cold Fusion platform, residing on the EPICORE centre secure web server, with SSL certificate and 128 bit encryption) will be used to permit real time balanced treatment allocation.

6.0 TREATMENT: BP MANAGEMENT PROTOCOLS

6.1 "Aggressive" BP Target (<140mmHg) Treatment Group

Patients randomized to the <140 mmHg group (n=135) will immediately receive a 10 mg IV bolus of labetalol, administered over 1 minute. A protocol designed to achieve and maintain systolic BP <140 mmHg within 60 minutes of randomization has been designed (Table 2). A key feature of this protocol is the utilization of IV enalapril, which can be given regularly (Q. 6 hourly), avoiding BP fluctuations, a problem which has been noted previously when using bolus-based protocols.[99] Patients randomized to the <140 mmHg group will be treated with 1.25 mg of IV enalapril immediately after labetalol administration. A lower limit of 120 mmHg has been stipulated, although given our experience in ICH ADAPT I, this is unlikely to be achieved. In the event of systolic BP falling below 120 mmHg, antihypertensive therapy will be held and patients will be fluid resuscitated with isotonic saline. Pressor agents will not be used.

6.2 "Conservative" BP Target (<180mmHg) Treatment Group

Patients randomized to the <180 mmHg group (n=135) will be administered parenteral antihypertensive therapy only if systolic BP is ≥180 mmHg (Table 3), consistent with current guidelines.[76]

6.3 Treatment Considerations

All patients will have continuous non-invasive BP and heart rate (HR) monitoring for a minimum of 24 h. BP and HR will be recorded most intensively during the hyperacute phase, as per the NINDS r-tPA protocol for vital signs monitoring. Antihypertensive drug use and dosages will be recorded concomitantly with BP and HR. Patients will be monitored regularly until study completion. We will document door-to-needle times with respect to the initiation of antihypertensive medication and the proportion of patients achieving BP targets within 1 hour of treatment.

At completion of the 24 h active treatment period, all patients will continue to receive standard stroke care and rehabilitation, and treating physicians will manage BP in the manner they feel is appropriate. Physicians will be encouraged to start oral antihypertensive therapy, administered via nasogastric feeding tube if necessary, on day 2. BP, HR and antihypertensive medication doses will continue to be monitored and recorded every 4 h for the first 48 h and then twice daily until discharge. Long-term goals for both patient groups after the active treatment period are a systolic BP of <140 mmHg, or <130 mmHg in those with diabetes, as per current stroke prevention and hypertension guidelines.[100]

6.4 Standard Stroke Care

With the exception of BP management, treatment of all ICH patients will be identical and consistent with current practice guidelines.[76, 101] This will include admission to a stroke unit in most cases and intensive care unit in those with decreased level of consciousness compromising airway protection. Oxygen is only necessary in patients with oximetry proven desaturation. Patients are kept NPO (nothing by mouth) until after swallowing assessment performed by trained speech language pathologists, and hydrated with 0.9% saline IV (no glucose). DVT prophylaxis includes compression stockings on admission and subcutaneous heparin (unfractionated or low molecular weight) after 72 h. Patients are mobilized by day 2, under the supervision of the physiotherapy and occupational therapy team. Fevers are treated with acetaminophen and infections aggressively managed. Given the lack of evidence for routine use, hyper-osmolar agents and mannitol are not recommended, but any administration will be recorded. All aspects of standard stroke unit care for 7 days after admission will be recorded.

6.5 Oral Anticoagulant Associated Sub-group

Anticoagulant-associated ICH (AAICH) accounts for about 12% of all ICH and is associated with higher rates of ICH expansion and worse clinical outcomes.[16] We have assessed treatment of warfarin-treated patients with two INR reversal strategies: Prothrombin Complex Concentrate (PCC) + Vitamin K and Fresh Frozen Plasma (FFP) + Vitamin K.[102] Although PCC results in more rapid correction of INR, ICH expansion rates are comparable to those seen in patients treated with FFP. Patients with INR values >1.5 will be reversed using a standardized protocol, utilizing

PCC where available. Door-to-needle treatment times for warfarin reversal will be documented as a quality assurance performance measure. In patients taking direct thrombin inhibitors (dabigatran) or Factor Xa antagonists (rivaroxaban or apixaban), the coagulopathy will be managed according to local practice. As no proven reversal strategy exists for any of these agents, a standardized protocol has not been developed, but all interventions will be recorded. Information about the characteristics and outcomes of ICH in patients taking these novel oral anticoagulants will be of special interest given their anticipated increased usage for patients with atrial fibrillation. By tracking consecutive ICH admissions, our study will allow comparison of incidence and outcomes between warfarin-associated ICH and those related to the newer anticoagulants. ICH expansion in AAICH patients will be assessed separately. It is important to include these patients, as they represent an increasing proportion of the ICH patient population. Furthermore, it is possible that anticoagulants and/or the underlying disease profile of these patients are relevant to DWI lesion pathogenesis. We hypothesize that DWI lesions will be more frequent in anticoagulated patients, reflecting their higher thromboembolic risk profile. The balanced randomization process will ensure an equal distribution of these patients in both treatment groups.

6.6 Surgical Management

Where an extraventricular drain is deemed to be required clinically, it will be placed *after* BP randomization. As part of vital sign assessment and stabilization, BP management is an emergent priority. If early surgical evacuation is planned immediately after the diagnostic CT scan, patients will not be enrolled. In cases where the decision to evacuate the hematoma is made after enrolment, a CT will be obtained immediately pre-operatively and this will be used as the follow-up scan for the purpose of assessing ICH growth (secondary endpoint). An MRI will be obtained post-operatively, but these patients will not be included in the primary endpoint analysis, given the confounding effects of surgery. The development of ischemic lesions in surgically treated patients is nonetheless relevant.

7.0 STUDY PROCEDURES AND EVALUATIONS

7.1 Clinical Screening/Baseline

Demographics, Glasgow Coma Scale (GCS) and National Institutes of Health Stroke Scale (NIHSS) scores (both of which are part of routine stroke patient assessment), time of symptom onset and diagnostic CT scan will all be recorded. If the CT scan is completed within 6 hours of onset and confirms evidence of a primary ICH, patients will be randomized. Stroke risk factors, past medical history and medications, with emphasis on antihypertensives, as well as standard clinical blood work (complete blood count and coagulation profile) will be recorded *after* randomization in order to avoid delays to BP treatment.

7.1.1 Consent Procedures:
The study will be initiated following approval of clinical trials from NCAT. Informed consent will be obtained from the patient, or substitute decision maker if the patient is incapacitated, prior to randomization. A participant information sheet will be provided at this time as well. Patients/caregivers will be free to withdraw from trial participation at any time and BP management will be continued as directed by the responsible physician.

7.2 Study Imaging Procedures

7.2.1 Baseline:
Immediately prior to randomization and BP reduction, patients will undergo a standard non-contrast CT diagnostic brain scan. This will consist of 5 mm slices, no gap (120 kvp, 300 mA per slice) through the entire brain (l8-20 slices with a 512x512 matrix). This scan is required to make the diagnosis of ICH and is not considered part of trial procedures. In the event of early neurological deterioration at any point, a repeat CT scan will be obtained immediately.

7.2.2 24-hour CT Scan:
All patients will have a repeat CT brain scan at 24±3 h, in order to assess for hematoma expansion and peri-hematoma edema volume. The scan parameters will be identical to that used in the diagnostic scan. A repeat CT scan at 24 hours is part of standard clinical care.[76]

7.2.3 48-hour MRI Scan (Primary Endpoint):
At 48±12 h, patients will undergo MRI scanning, including a T1-weighted sagittal localizer, DWI, Gradient Recalled Echo (GRE)/Susceptibility Weighted Imaging (SWI), diffusion-weighted (DWI) and perfusion-weighted images (PWI). Single shot Echoplanar (EPI) DWI images obtained using diffusion gradient strengths (b values) between 0 s/mm2, equivalent to a T2-weighted image, and 1000 s/mm2, applied in 3 orthogonal planes, will be obtained. These will be combined to form isotropic (trace) diffusion images and Apparent Diffusion Coefficient (ADC) maps will be generated from these raw data. DWI acquisition parameters will be: repetition time (TR) of 3 s, spin echo time (TE) of 86 ms, 8 averages, 128x128 matrix base resolution zero filled to 256x256, 22 cm field-of-view, and 1396 Hz/pixel acquisition bandwidth. The entire brain will be imaged using 19-24 contiguous axial slices each 5 mm in thickness, with a 1.5 mm inter-slice gap.

Perfusion images will be derived from the concentration-time curve obtained after the administration of intravenous gadolinium (gadopentate (Magnevist™) 0.5 mmol/ml, 15 ml; 5 ml/sec power injection via an 18 g angiocatheter in an antecubital vein, followed by 20 ml saline flush at the same rate) with EPI gradient-echo (T2*) images acquired every 1.2 seconds for 80 seconds (19 axial slices at each time point). Nephrogenic

systemic fibrosis associated with gadolinium exposure has been reported only in patients with pre-existing severe renal failure.[103] As a precaution, patients with estimated Glomerular Filtration Rate (eGFR) <30 ml/min (Cockcroft-Gault equation), PWI will not be performed. In patients with moderate renal impairment (estimated eGFR 30-60 ml/min), a lower volume of concentrated gadolinium (gadobutrol (Gadovist™), 1.0 mmol/ml, 7.5 ml) will be used. Patients with eGFR<30 ml/min will not undergo PWI. These sequences can be completed in 30 minutes.

DWI will be used to assess the primary endpoint. Phase contrast Magnetic Resonance Angiogram (MRA) will be obtained to determine the presence of intra-arterial occlusions, which may predispose to ischemic injury. PWI data will be used to generate CBF maps in order to determine if DWI lesions are correlated with hypoperfusion. GRE/SWI sequences will be used to assess the burden (number, total volume and topography) of cerebral microbleeds. PWI data will also be used to generate maps of blood-brain-barrier leakage, which may be relevant to DWI lesion development.

7.2.4 Day 7 MRI Scan (Secondary Endpoint):

A repeat MRI will be obtained at 7 ± 2 days to assess for new DWI lesion development and evolution of those identified at 24 hours. This is a secondary endpoint.

7.2.5 Day 30 MRI Scan (Secondary Endpoint):

A repeat MRI will be obtained at 30 ± 5 days to assess for new DWI lesion development and evolution of those identified at 24 hours and 7 days. Secondary endpoints of perihematoma edema growth will also be assessed at this time. All sequences will be identical to those at Day 7, but PWI will not be performed. This is a secondary endpoint.

7.3 Clinical Follow-up Assessments

7.3.1 In Hospital:

In addition to BP data, GCS and NIHSS scores will be collected in the event of early neurological deterioration. Both of these scores will also be recorded at the time of each MRI scan and at hospital discharge or transfer to alternate level of care, i.e. rehabilitation or long-term care facility. Discharge modified Rankin Scores (mRS) will also be recorded. Cognitive changes will be assessed with the Montreal Cognitive Assessment (MoCA) at the time of each MRI scan. It is important to assess cognition, as ICH is a major cause of cognitive impairment and dementia that has received little attention in other trials. We also hypothesize that MoCA will be a sensitive marker of the clinical impact of DWI lesions.

7.3.2 Day 30 Assessments:
Early mortality in ICH studies has traditionally been defined as death within 30 days of onset. A standardized interview aimed at determining mortality and current residence of the patient (home/hospital/rehabilitation hospital/long-term care facility) will be administered at the time of the day 30 MRI. The NIHSS and MoCA scores will also be recorded, as will modified Rankin scale (mRS) scores. The latter are the standard disability assessment in stroke trials, ranging from 0 (no symptoms) to 1 (symptoms; no disability), 2 (mild disability), 3 (moderate disability; independent), 4 (dependent), 5 (severe disability, bedridden, incontinent), 6 (death).[104, 105] Quality of life will be assessed with the EQ-5D.[106]

7.3.3 Day 90 Assessments:
This is the standard time point for measuring functional outcomes in stroke trials, as the bulk of neurological recovery occurs within that time frame. All neurological, functional and cognitive disability tests will be repeated at this time.

As a clinical procedure, we will record where the patient discharged to (place of residence, rehabilitation hospital, long-term care facility), and only patients who can not come to the clinic will be contacted by phone. In order to avoid calling carers/relatives of deceased participants for the 30-day and the 90-day follow-up assessment, the following actions will be taken before the call is made:
1; Contact the rehabilitation hospital / long-term care facility;
2; Check the electronic medical record (EMR);
3; Contact the treating neurologist and/or the GP of the patient;
4; Search on The Ryerson Index for death notices;

8.0 RETROSPECTIVE STUDY AT POWH

In order to determine current blood pressure management trends in this hospital, we will conduct a retrospective chart review, using the EMR and PACS as data sources. We will conduct a two-year chart review of all patients admitted with a diagnosis of intracerebral haemorrhage, including those cared for by Neurology and Neurosurgery. We will access images on the PACS system/Enterprise Imaging Repository in order to measure hematoma volumes and assess for expansion when serial imaging was completed.

9.0 STATISTICAL ANALYSIS AND POWER CALCULATION

The primary endpoint of ischemic lesion rates will be tested using a Fisher's Exact test. The sample size is based on an estimated ischemic lesion frequency of 26% in the <180 mmHg target group. This is based on the mean observed rate of DWI lesions in the six studies

published to date (*Table 1*). Menon et al reported the odds ratio for DWI lesion occurrence was 1.03 for each decrease in mmHg MAP between baseline and the MRI scan.[80] In ICH ADAPT I, the MAP in the <150 mmHg treatment group decreased by an average of 28 mmHg at the time of the primary endpoint assessment. Assuming a similar treatment effect in ICH ADAPT II, the predicted effect of BP reduction to <140 mmHg systolic is an odds ratio of DWI lesion incidence of 1.84. The trial has therefore been powered to detect a 0.84 (relative) increase in the frequency of DWI lesions in the <140 mmHg target group. *The predicted absolute increase in the proportion of patients with DWI lesions will be 22%.* The hypotheses statements are:

H_0: the proportion of patients with DWI lesions in the <140 mmHg treatment arm will be ≤ 0.48.
H_A: the proportion of patients with DWI lesions in the <140 mmHg treatment arm will be > 0.48.

Although it is possible that aggressive BP reduction will be associated with *decreased* DWI lesion frequency, the outcome of this study is primarily of interest if the direction of change is towards increased DWI lesion frequency, i.e. harm. The primary analysis will therefore be a one-sided test of proportions at the alpha = 0.025 level. There will be no interim analyses for efficacy or futility. A sample size of 180 evaluable patients will be required to reject the null hypothesis that BP reduction does not result in more frequent DWI lesion development with 80% power (alpha=0.025). To account for attrition, cross-over, withdrawal of consent and missing data, we will inflate the sample size by 33% (n=270 total). This relatively large number is required, as many patients will have contraindications to MRI, have died or be too medically unstable to undergo MRI at 48 hours. The total target sample size will be N=270 patients, which is feasible at our participating sites.

10.0 CENTRAL IMAGING AND ADJUDICATION OF ANALYSIS

All imaging data will be anonymized completed at POWH under the supervision of Professor Butcher, who has 19 years experience with the image processing techniques. All measurements will be made by central raters blinded to BP treatment group allocation and clinical data. Upon trial completion, images will be made available to the wider stroke community, in order to test additional hypotheses related to hematoma/perihematoma edema evolution.

Standard 3-dimensional volumes will be created from raw CT and MRI data using purpose built CT analysis software (MiStar, Apollo Medical Imaging, Melbourne).[107] Using standard planimetric techniques, hematoma volumes will be calculated on CT images. The absolute change in hematoma volumes as well as the proportion with significant expansion (≥ 6 ml) from baseline to 24 h will be calculated. Intraventricular hemorrhage (IVH) and perihematoma volumes will also be measured planimetrically. MRI data will be analysed using planimetric techniques as well. DWI sequences will be assessed for the presence, number and total volume of regions with diffusion restriction. Apparent Diffusion Coefficient Maps will be used to ensure

all lesions represent true diffusion restriction and not T2 shine through effects. FLAIR sequences will also be utilized to assess chronic small vessel ischemic changes (*Figure* 7). Periventricular white matter hyperintensity volumes will be measured using advanced intensity threshold and planimetric techniques provided in the Quantomo package.

11.0 TRIAL MANAGEMENT

11.1 Coordinating Centre

The ICH ADAPT II data management centre is located within the Epidemiology Coordinating and Research (EPICORE) Centre (www.epicore.ualberta.ca). This is a comprehensive clinical research organization within the Department of Medicine at the University of Alberta, with extensive expertise in conducting large multicentre international trials. EPICORE employs expert project management and statistical analysis staff, directed by Dr. R. Tsuyuki. A web-based (SSL certificate and 128 bit encryption) case report form (developed with Microsoft SQL and Visual Studio.net) has been designed by EPICORE to facilitate data entry and analysis. Data entries will be monitored daily and queries generated by EPICORE staff. De-identified clinical and imaging data obtained from the POWH will be shared with the Coordinating Centre via the web-based case report form.

12.0 DATA SAFETY MONITORING BOARD

Anonymized data (including clinical and imaging data) will be sent to an independent data safety monitoring board (DSMB) every six month for review. The DSMB is chaired by Dr. Georgios Tsivgoulis. The committee will review a) the proportion of patients with worsening by ≥4 points on the NIHSS within 48 h and b) 90-day mortality after 33% and 67% enrolment. The committee may modify/stop the trial at any point.

13.0 ADVERSE EVENT REPORTING

Investigators will report all serious adverse events (SAE), using standardized event, resolution and association codes. The SAE reporting period (48 h) includes the entire BP treatment duration and an additional 24 h. SAEs of interest include neurological deterioration, defined as an increase in NIHSS of ≥4 points and severe hypotension (systolic BP <100 mmHg).

14.0 ADDITIONAL INFORMATION

14.1 Feasibility

Patients presenting with suspected acute stroke within 6 hours of onset are in the thrombolysis/intra-arterial clot retrieval therapeutic time window. For this reason, Emergency Department triage staff at all stroke/telestroke centres have ‘code stroke’ protocols to alert the

24/7 on call stroke teams immediately as these patients arrive at the hospital (pre-hospital protocols include alerting the stroke team prior to patient arrival). ICH patients are therefore scanned with CT (available 24 hours) and assessed quickly by stroke team staff, facilitating rapid randomization and enrolment in ICH ADAPT II. At the University of Alberta Hospital, approximately 75 ICH patients are assessed acutely on an annual basis.[77] The median time from symptom onset to diagnostic CT in our retrospective analysis was 3.3 hours.[77] In ICH ADAPT I, the median time from symptom onset to hospital presentation was 1.9 h and median time to CT was 2.4 h (door to CT scan = 30 min). Delays in enrolment were due to the need for consent prior to the much more complex imaging studies obtained as part of that trial. Based on the number of participating sites, we conservatively expect to enrol 90 patients per year across all sites and approximately 10 patients annually at POWH. All participating sites already use MRI to study ischemic stroke. Scans will be performed on standard clinical magnets, on a contractual basis.

14.2 Significance and Clinical Impact

Equipoise persists with respect to optimal management of BP after ICH. Recent evidence of ischemic injury in ICH patients has renewed calls for a more conservative approach to acute hypertension. At the same time, the lack of a convincing treatment effect in INTERACT II has not led to an immediate change in management guidelines or treatment effects. It is possible that there may be no net clinical benefit of BP reduction, potentially related to increased frequency of ischemic injury. Trials with physiological endpoints, such as ICH ADAPT II, are more sensitive to injury in individual patients, which may not be evident in a larger clinical endpoint study. Our findings will provide definitive evidence for the link, or lack thereof, between BP reduction and ischemic injury. This will add support for early BP reduction, or alternatively result in a more nuanced approach to acute hypertension management. Our study will also be the first to prospectively identify other risk factors for ischemic injury after ICH.

15.0 DATA HANDLING AND RECORD KEEPING

Data obtained from POWH will be recorded in an electronic CRF, which will be kept on a SESLHD ICT operated virtual server. Access to the imaging and clinical databases will be secure and password protected, and managed by the investigators, Professor Butcher and Dr. Wilson, both of whom are employees of the POWH, or staff working directly under their supervision. Professor Butcher is responsible to the data management. Any information used for publication / presentation / collaboration will be de-identified. All information will be kept private, confidential and secure.

The study was funded by a grant held by Dr. Butcher from the Heart and Stroke Foundation of Canada, and it is started in 2014. All infrastructure for the trial, including the web-based randomisation site and the REDCAP (a secure web application for building and managing online surveys and databases) database are currently operating at the University of Alberta. Dr. Butcher remains the custodian of those data, as he is an Adjunct Professor at the University of

Alberta. Therefore, de-identified data (including clinical and imaging data) will also be entered into the REDCAP clinical database at the University of Alberta. At the completion of the trial, the complete database will be analysed by Dr. Butcher's research team at the University of New South Wales, with assistance from his collaborators at the University of Alberta.

Approval from Professor Butcher is required to access to the anonymized data. The data are the property of POWH and UNSW. Management of the data will be the responsibility of Professor Butcher, in the event of his departure, control of the data will be transferred to his successor, with the approval of the Head of the Prince of Wales Clinical School and the Neurosciences, Cancer and Rehabilitation Director at POWH.

It is likely ***that our*** clinical and imaging ***data will be requested by other groups internationally once the study is complete. We share only composite data, or individual patient data that has been completely de-identified.***

16.0 ETHICAL CONSIDERATIONS

This study will be conducted according to the Notes for Guidance on Good Clinical Practice as adopted by the Australian Therapeutic Goods Administration (2000) (CPMP/ICH/135/95) and the ICH GCP Guidelines. Applicable government regulations and University of New South Wales research policies and procedures will also be followed.

This protocol and any amendments will be submitted to the University of New South Wales Human Research Ethics Committee for formal approval to conduct the study. The decision of the research ethics boards concerning the conduct of the study will be made in writing to the investigator.

All subjects for this study will be provided a consent form describing this study and providing sufficient information for subjects to make an informed decision about their participation in this study. This consent form will be submitted with the protocol for review and approval by the research ethics boards. The formal consent of a subject, using the HREC consent form, will be obtained before that subject is subjected to any study procedure. This consent form must be signed by the subject, or legally acceptable surrogate, as well as the investigator or investigator-designated research professional obtaining the consent.

REFERENCES

1. Butcher K, Laidlaw J. Current intracerebral haemorrhage management. *J Clin Neurosci*. 2003;10:158-167
2. Qureshi AI, Tuhrim S, Broderick JP, Batjer HH, Hondo H, Hanley DF. Spontaneous intracerebral hemorrhage. *N Engl J Med*. 2001;344:1450-1460
3. Butcher K, Baird T, Parsons M, Davis S. Medical management of intracerebral hemorrhage. *Neurosurgery Quarterly*. 2002;12:261-278
4. Bamford J, Sandercock P, Dennis M, Burn J, Warlow C. A prospective study of acute cerebrovascular disease in the community: The oxfordshire community stroke project--1981-86. 2. Incidence, case fatality rates and overall outcome at one year of cerebral infarction, primary intracerebral and subarachnoid haemorrhage. *J Neurol Neurosurg Psychiatry*. 1990;53:16-22
5. Broderick JP, Brott T, Tomsick T, Huster G, Miller R. The risk of subarachnoid and intracerebral hemorrhages in blacks as compared with whites. *N Engl J Med*. 1992;326:733-736
6. Thrift AG, Donnan GA, McNeil JJ. Epidemiology of intracerebral hemorrhage. *Epidemiol Rev*. 1995;17:361-381
7. Brown RD, Whisnant JP, Sicks JD, O'Fallon WM, Wiebers DO. Stroke incidence, prevalence, and survival. Secular trends in rochester, minnesota, through 1989. *Stroke*. 1996;27:373-380
8. Mayo NE, Neville D, Kirkland S, Ostbye T, Mustard CA, Reeder B, et al. Hospitalization and case-fatality rates for stroke in canada from 1982 through 1991: The canadian collaborative study group of stroke hospitalizations1. *Stroke*. 1996;27:1215-1220
9. Foulkes M, Wolf P, Price T, Mohr J, Hier D. The stroke data bank: Design, methods, and baseline characteristics. *Stroke*. 1988;19:547-554
10. Hart RG, Boop BS, Anderson DC. Oral anticoagulants and intracranial hemorrhage : Facts and hypotheses. *Stroke*. 1995;26:1471-1477
11. Juvela S. Prevalence of risk factors in spontaneous intracerebral hemorrhage and aneurysmal subarachnoid hemorrhage. *Arch. Neurol.* 1996;53:734-740
12. Broderick JP. *Handbook of neuroepidemiology*. New York: Marcel Decker Inc; 1994.
13. Counsell C, Boonyakarnkul S, Dennis M. Primary intracerebral haemorrhage in the oxfordshire community stroke project, 2: Prognosis. *Cerebrovasc Dis*. 1995;5:26-34
14. Dennis MS, Burn JPS, Sandercock PAG, Bamford JM, Wade DT, Warlow CP. Long-term survival after first-ever stroke: The oxfordshire community stroke project. *Stroke*. 1993;24:796-800
15. Broderick JP. Natural history of primary intracerebral hemorrhage. In: Whisnant JP, ed. *Stroke: Populations, cohorts, and clinical trials*. Oxford: Butterworth-Heinemann; 1993:154-173.
16. Gladstone DJ, Rodan LH, Hill MD, Fang J, Linday P, Silver FL, et al. Intracerebral hemorrhage in patients taking warfarin compared to non-anticoagulated patients: Results from the registry of the canadian stroke network. *Canadian Journal of Neurological Sciences*. 2011 (In Press)

17. Mendelow AD, Gregson BA, Fernandes HM, Murray GD, Teasdale GM, Hope DT, et al. Early surgery versus initial conservative treatment in patients with spontaneous supratentorial intracerebral haematomas in the international surgical trial in intracerebral haemorrhage (stich): A randomised trial. *Lancet*. 2005;365:387-397
18. Bauer RB, Tellez H. Dexamethasone as treatment in cerebrovascular disease, 2: A controlled study in acute cerebral infarction. *Stroke*. 1973;4:547-555
19. Poungvarin N, Bhoopat W, Viriyavejakul A, Rodprasert P, Buranasiri P, Sukondhabhant S, et al. Effects of dexamethasone in primary supratentorial intracerebral hemorrhage. *N Engl J Med*. 1987;316:1229-1233
20. Yu YL, Kumana CR, Lauder IJ, Cheung YK, Chan FL, Kou M, et al. Treatment of acute cerebral hemorrhage with intravenous glycerol: A double-blind, placebo-controlled, randomized trial. *Stroke*. 1992;23:967-971
21. Haemodilution in acute stroke: Results of the italian haemodilution trial. Italian acute stroke study group. *Lancet*. 1988;1:318-321
22. Bogousslavsky J, Van Melle G, Regli F. The lausanne stroke registry: Analysis of 1,000 consecutive patients with first stroke. *Stroke*. 1988;19:1083-1092
23. Lang EW, Ren Ya Z, Preul C, Hugo HH, Hempelmann RG, Buhl R, et al. Stroke pattern interpretation: The variability of hypertensive versus amyloid angiopathy hemorrhage. *Cerebrovasc Dis*. 2001;12:121-130.
24. Fisher CM. Pathological observations in hypertensive cerebral hemorrhage. *J Neuropathol Exp Neurol*. 1971;30:536-550
25. Broderick JP. Volume of intracerebral hemorrhage. A powerful and easy-to-use predictor of 30-day mortality. *Stroke*. 1993;24:987-993
26. Helweg-Larsen S, Sommer W, Strange P, Lester P, Boysen G. Prognosis for patients treated conservatively for spontaneous intracerebral hematomas. *Stroke*. 1984;15:1045-1048
27. Auer RN, Sutherland GR. Primary intracerebral hemorrhage: Pathophysiology. *Can J Neurol Sci*. 2005;32 Suppl 2:S3-12
28. Sutherland GR, Auer RN. Primary intracerebral hemorrhage. *J Clin Neurosci*. 2006;13:511-517
29. Fujii Y, Tanaka R, Takeuchi S, Koike T, Minakawa T, Sasaki O. Hematoma enlargement in spontaneous intracerebral hemorrhage. *J Neurosurg*. 1994;80:51-57
30. Kazui S, Naritomi H, Yamamoto H, Sawada T, Yamaguchi T. Enlargement of spontaneous intracerebral hemorrhage: Incidence and time course. *Stroke*. 1996;27:1783-1787
31. Brott T, Broderick J, Kothari R, Barsan W, Tomsick T, Sauerbeck L, et al. Early hemorrhage growth in patients with intracerebral hemorrhage. *Stroke*. 1997;28:1-5
32. Brott T, Broderick J, Kothari R, Barsan W, Tomsick T, Sauerbeck L, et al. Early hemorrhage growth in patients with intracerebral hemorrhage. *Stroke*. 1997;28:1-5
33. Kazui S, Minematsu K, Yamamoto H, Sawada T, Yamaguchi T. Predisposing factors to enlargement of spontaneous intracerebral hematoma. *Stroke*. 1997;28:2370-2375
34. Kazui S, Naritomi H, Yamamoto H, Sawada T, Yamaguchi T. Enlargement of spontaneous intracerebral hemorrhage: Incidence and time course. *Stroke*. 1996;27:1783-1787

35. Fujii Y, Takeuchi S, Sasaki O, Minakawa T, Tanaka R. Multivariate analysis of predictors of hematoma enlargement in spontaneous intracerebral hemorrhage. *Stroke*. 1998;29:1160-1166
36. Brott T, Thalinger K, Hertzberg V. Hypertension as a risk factor for spontaneous intracerebral hemorrhage. *Stroke*. 1986;17:1078-1083
37. Thrift AG, McNeil JJ, Forbes A, Donnan GA. Risk factors for cerebral hemorrhage in the era of well-controlled hypertension. Melbourne risk factor study (merfs) group. *Stroke*. 1996;27:2020-2025.
38. Thrift AG, McNeil JJ, Forbes A, Donnan GA. Three important subgroups of hypertensive persons at greater risk of intracerebral hemorrhage. Melbourne risk factor study group. *Hypertension*. 1998;31:1223-1229.
39. Staessen JA, Fagard R, Thijs L, Celis H, Arabidze GG, Birkenhager WH, et al. Randomised double-blind comparison of placebo and active treatment for older patients with isolated systolic hypertension. The systolic hypertension in europe (syst-eur) trial investigators. *Lancet*. 1997;350:757-764
40. Collins R, Peto R, MacMahon S, Hebert P, Fiebach NH, Eberlein KA, et al. Blood pressure, stroke, and coronary heart disease. Part 2, short-term reductions in blood pressure: Overview of randomised drug trials in their epidemiological context. *Lancet*. 1990;335:827-838
41. MacMahon S, Peto R, Cutler J, Collins R, Sorlie P, Neaton J, et al. Blood pressure, stroke, and coronary heart disease. Part 1, prolonged differences in blood pressure: Prospective observational studies corrected for the regression dilution bias. *Lancet*. 1990;335:765-774
42. Group SCR. Prevention of stroke by antihypertensive drug treatment in older persons with isolated systolic hypertension. Final results of the systolic hypertension in the elderly program (shep). *JAMA*. 1991;265:3255-3264
43. Collins R, MacMahon S. Blood pressure, antihypertensive drug treatment and the risks of stroke and of coronary heart disease. *Br Med Bull*. 1994;50:272-298
44. Blood Pressure Lowering Treatment Trialists' Collaboration. Effects of different blood pressure lowering regimens on major cardiovascular events: Second cycle of prospectively designed overviews. *Lancet*. 2003;362:1527-1535
45. Perry HM, Jr., Davis BR, Price TR, Applegate WB, Fields WS, Guralnik JM, et al. Effect of treating isolated systolic hypertension on the risk of developing various types and subtypes of stroke: The systolic hypertension in the elderly program (shep). *JAMA*. 2000;284:465-471
46. Group PC. Randomised trial of a perindopril-based blood-pressure-lowering regimen among 6,105 individuals with previous stroke or transient ischaemic attack. *Lancet*. 2001;358:1033-1041
47. Wallace JD, Levy LL. Blood pressure after stroke. *JAMA*. 1981;246:2177-2180
48. Harper G, Castleden CM, Potter JF. Factors affecting changes in blood pressure after acute stroke. *Stroke*. 1994;25:1726-1729
49. Ong TZ, Raymond AA. Risk factors for stroke and predictors of one-month mortality. *Singapore Med J*. 2002;43:517-521

50. Fogelholm R, Avikainen S, Murros K. Prognostic value and determinants of first-day mean arterial pressure in spontaneous supratentorial intracerebral hemorrhage. *Stroke*. 1997;28:1396-1400
51. Portenoy RK, Lipton RB, Berger AR, Lesser ML, Lantos G. Intracerebral haemorrhage: A model for the prediction of outcome. *J Neurol Neurosurg Psychiatry*. 1987;50:976-979
52. Tuhrim S, Dambrosia JM, Price TR, Mohr JP, Wolf PA, Heyman A, et al. Prediction of intracerebral hemorrhage survival. *Ann Neurol*. 1988;24:258-263
53. Broderick JP, Brott TG, Duldner JE, Tomsick T, Huster G. Volume of intracerebral hemorrhage: A powerful and easy-to-use predictor of 30-day mortality. *Stroke*. 1993;24:987-993
54. Carlberg B, Asplund K, Hagg E. The prognostic value of admission blood pressure in patients with acute stroke. *Stroke*. 1993;24:1372-1375
55. Dandapani BK, Suzuki S, Kelley RE, Reyes-Iglesias Y, Duncan RC. Relation between blood pressure and outcome in intracerebral hemorrhage. *Stroke*. 1995;26:21-24
56. Leira R, Dávalos A, Silva Y, Gil-Peralta A, Tejada J, Garcia M, et al. Early neurologic deterioration in intracerebral hemorrhage: Predictors and associated factors. *Neurology*. 2004;63:461-467
57. Qureshi AI, Safdar K, Weil J, Barch C, Bliwise DL, Colohan AR, et al. Predictors of early deterioration and mortality in black americans with spontaneous intracerebral hemorrhage. *Stroke*. 1995;26:1764-1767
58. Terayama Y, Tanahashi N, Fukuuchi Y, Gotoh F. Prognostic value of admission blood pressure in patients with intracerebral hemorrhage: Keio cooperative stroke study. *Stroke*. 1997;28:1185-1188
59. Meyer JS, Bauer RB. Medical treatment of spontaneous intracranial hemorrhage by the use of hypotensive drugs. *Neurology*. 1962;12:36-47
60. Dunne JW, Chakera T, Kermode S. Cerebellar haemorrhage, diagnosis and treatment: A study of 75 consecutive cases. *QJM*. 1987;64:739-754
61. Qureshi AI, Bliwise DL, Bliwise NG, Akbar MS, Uzen G, Frankel MR. Rate of 24-hour blood pressure decline and mortality after spontaneous intracerebral hemorrhage: A retrospective analysis with a random effects regression model. *Crit Care Med*. 1999;27:480-485
62. Okumura K, Ohya Y, Maehara A, Wakugami K, Iseki K, Takishita S. Effects of blood pressure levels on case fatality after acute stroke. *J Hypertens*. 2005;23:1217-1223
63. Carhuapoma JR, Ulatowski JA. Blood pressure control after intracerebral hemorrhage: Have we reached the target? *Crit Care Med*. 2006;34:2023-2024
64. Ohwaki K, Yono E, Nagashima H, Hirata M, Nakagomi T, Tamura A. Blood pressure management in acute intracerebral hemorrhage: Relationship between elevated blood pressure and hematoma enlargement. *Stroke*. 2004;35:1353-1367
65. Takizawa K, Suzuki A, Nagate K, Kawamura S, Sato M, Yasui N, et al. Blood pressure control in acute stages of hypertensive intracerebral hemorrhage to prevent growth of hematoma. In: Yasui N, ed. *Brain hemorrhage '99*. Tokyo: NEURON publishing; 2002:11-18.
66. Anderson CS, Huang Y, Arima H, Heeley E, Skulina C, Parsons MW, et al. Effects of early intensive blood pressure-lowering treatment on the growth of hematoma and

perihematomal edema in acute intracerebral hemorrhage: The intensive blood pressure reduction in acute cerebral haemorrhage trial (interact). *Stroke*. 2010;41:307-312

67. Qureshi AI, Palesch YY, Martin R, Novitzke J, Cruz-Flores S, Ehtisham A, et al. Effect of systolic blood pressure reduction on hematoma expansion, perihematomal edema, and 3-month outcome among patients with intracerebral hemorrhage: Results from the antihypertensive treatment of acute cerebral hemorrhage study. *Archives of neurology*. 2010;67:570-576
68. Delcourt C, Huang Y, Wang J, Heeley E, Lindley R, Stapf C, et al. The second (main) phase of an open, randomised, multicentre study to investigate the effectiveness of an intensive blood pressure reduction in acute cerebral haemorrhage trial (interact2). *Int J Stroke*. 2010;5:110-116
69. Qureshi AI, Palesch YY. Antihypertensive treatment of acute cerebral hemorrhage (atach) ii: Design, methods, and rationale. *Neurocritical care*. 2011
70. Mendelow AD. Mechanisms of ischemic brain damage with intracerebral hemorrhage. *Stroke*. 1993;24:I115-117; discussion I118-119
71. Adams RE, Powers WJ. Management of hypertension in acute intracerebral hemorrhage. *Crit Care Clin*. 1997;13:131-161
72. Mayer SA, Thomas CE, Diamond BE. Asymmetry of intracranial hemodynamics as an indicator of mass effect in acute intracerebral hemorrhage: A transcranial doppler study. *Stroke*. 1996;27:1788-1792
73. Broderick JP, Adams HP, Jr., Barsan W, Feinberg W, Feldmann E, Grotta J, et al. Guidelines for the management of spontaneous intracerebral hemorrhage: A statement for healthcare professionals from a special writing group of the stroke council, american heart association. *Stroke*. 1999;30:905-915
74. Hacke W, Kaste M, Bogousslavsky J, Brainin M, Chamorro A, Lees K, et al. European stroke initiative recommendations for stroke management: Update 2003. *Cerebrovasc Dis*. 2003;16:311-337
75. Bath P, Chalmers J, Powers W, Beilin L, Davis S, Lenfant C, et al. International society of hypertension (ish): Statement on the management of blood pressure in acute stroke. *J Hypertens*. 2003;21:665-672
76. Morgenstern LB, Hemphill JC, 3rd, Anderson C, Becker K, Broderick JP, Connolly ES, Jr., et al. Guidelines for the management of spontaneous intracerebral hemorrhage: A guideline for healthcare professionals from the american heart association/american stroke association. *Stroke*. 2010;41:2108-2129
77. Manawadu D, Jeerakathil T, Roy A, Orwaard-Wong K, Butcher K. Blood pressure management in acute intracerebral haemorrhage guidelines are poorly implemented in clinical practice. *Clin Neurol Neurosurg*. 2010;112:858-864
78. Butcher K, Jeerakathil T, Emery D, Dowlatshahi D, Hill MD, Sharma M, et al. The intracerebral haemorrhage acutely decreasing arterial pressure trial: Ich adapt. *Int J Stroke*. 2010;5:227-233
79. Anderson CS, Heeley E, Huang Y, Wang J, Stapf C, Delcourt C, et al. Rapid blood-pressure lowering in patients with acute intracerebral hemorrhage. *The New England journal of medicine*. 2013

80. Menon RS, Burgess RE, Wing JJ, Gibbons MC, Shara NM, Fernandez S, et al. Predictors of highly prevalent brain ischemia in intracerebral hemorrhage. *Annals of neurology*. 2012;71:199-205
81. Prabhakaran S, Naidech AM. Ischemic brain injury after intracerebral hemorrhage: A critical review. *Stroke; a journal of cerebral circulation*. 2012;43:2258-2263
82. Garg RK, Liebling SM, Maas MB, Nemeth AJ, Russell EJ, Naidech AM. Blood pressure reduction, decreased diffusion on mri, and outcomes after intracerebral hemorrhage. *Stroke; a journal of cerebral circulation*. 2012;43:67-71
83. Gregoire SM, Charidimou A, Gadapa N, Dolan E, Antoun N, Peeters A, et al. Acute ischaemic brain lesions in intracerebral haemorrhage: Multicentre cross-sectional magnetic resonance imaging study. *Brain : a journal of neurology*. 2011;134:2376-2386
84. Prabhakaran S, Gupta R, Ouyang B, John S, Temes RE, Mohammad Y, et al. Acute brain infarcts after spontaneous intracerebral hemorrhage: A diffusion-weighted imaging study. *Stroke; a journal of cerebral circulation*. 2010;41:89-94
85. Kang DW, Han MK, Kim HJ, Yun SC, Jeon SB, Bae HJ, et al. New ischemic lesions coexisting with acute intracerebral hemorrhage. *Neurology*. 2012;79:848-855
86. Arsava EM, Kayim-Yildiz O, Oguz KK, Akpinar E, Topcuoglu MA. Elevated admission blood pressure and acute ischemic lesions in spontaneous intracerebral hemorrhage. *Journal of stroke and cerebrovascular diseases : the official journal of National Stroke Association*. 2013;22:250-254
87. Butcher K, Emery D. Acute stroke imaging. Part i: Fundamentals. *Can J Neurol Sci*. 2010;37:4-16
88. Butcher K, Emery D. Acute stroke imaging. Part ii: The ischemic penumbra. *Can J Neurol Sci*. 2010;37:17-27
89. Butcher K, Baird T, MacGregor L, Desmond P, Tress B, Davis S. Perihematomal edema in primary intracerebral hemorrhage is plasma derived. *Stroke*. 2004;35:1879-1885
90. Olivot JM, Mlynash M, Kleinman JT, Straka M, Venkatasubramanian C, Bammer R, et al. Mri profile of the perihematomal region in acute intracerebral hemorrhage. *Stroke; a journal of cerebral circulation*. 2010;41:2681-2683
91. Singer OC, Kurre W, Humpich MC, Lorenz MW, Kastrup A, Liebeskind DS, et al. Risk assessment of symptomatic intracerebral hemorrhage after thrombolysis using dwi-aspects. *Stroke; a journal of cerebral circulation*. 2009;40:2743-2748
92. Murat Arsava E, Kayim-Yildiz O, Oguz KK, Akpinar E, Topcuoglu MA. Elevated admission blood pressure and acute ischemic lesions in spontaneous intracerebral hemorrhage. *Journal of stroke and cerebrovascular diseases : the official journal of National Stroke Association*. 2011
93. Kimberly WT, Gilson A, Rost NS, Rosand J, Viswanathan A, Smith EE, et al. Silent ischemic infarcts are associated with hemorrhage burden in cerebral amyloid angiopathy. *Neurology*. 2009;72:1230-1235
94. Brazzelli M, Sandercock PA, Chappell FM, Celani MG, Righetti E, Arestis N, et al. Magnetic resonance imaging versus computed tomography for detection of acute vascular lesions in patients presenting with stroke symptoms. *Cochrane Database Syst Rev*. 2009:CD007424

95. Kothari RU, Brott T, Broderick JP, Barsan WG, Sauerbeck LR, Zuccarello M, et al. The abcs of measuring intracerebral hemorrhage volumes. *Stroke; a journal of cerebral circulation*. 1996;27:1304-1305
96. Hemphill JC, 3rd, Farrant M, Neill TA, Jr. Prospective validation of the ich score for 12-month functional outcome. *Neurology*. 2009;73:1088-1094
97. Clarke JL, Johnston SC, Farrant M, Bernstein R, Tong D, Hemphill JC, 3rd. External validation of the ich score. *Neurocritical care*. 2004;1:53-60
98. Hemphill JC, 3rd, Bonovich DC, Besmertis L, Manley GT, Johnston SC. The ich score: A simple, reliable grading scale for intracerebral hemorrhage. *Stroke; a journal of cerebral circulation*. 2001;32:891-897
99. Qureshi AI, Mohammad YM, Yahia AM, Suarez JI, Siddiqui AM, Kirmani JF, et al. A prospective multicenter study to evaluate the feasibility and safety of aggressive antihypertensive treatment in patients with acute intracerebral hemorrhage. *J Intensive Care Med*. 2005;20:34-42
100. Guidelines Subcommittee. 1999 world health organization - international society of hypertension guidelines for the management of hypertension. *J Hypertens*. 1999;17:151-183
101. Lindsay P, Bayley M, McDonald A, Graham ID, Warner G, Phillips S. Toward a more effective approach to stroke: Canadian best practice recommendations for stroke care. *CMAJ*. 2008;178:1418-1425
102. Asdaghi N, Poon C, Jeerakathil T, Butcher K. Hematoma expansion in anticogulant associated intracerebral hemorrhage despite prothrombin complex concentrate treatment. *International Stroke Conference*. 2011;42:e220
103. Lim YL, Lee HY, Low SC, Chan LP, Goh NS, Pang SM. Possible role of gadolinium in nephrogenic systemic fibrosis: Report of two cases and review of the literature. *Clin. Exp. Dermatol.* 2007;32:353-358
104. Bruno A, Akinwuntan AE, Lin C, Close B, Davis K, Baute V, et al. Simplified modified rankin scale questionnaire: Reproducibility over the telephone and validation with quality of life. *Stroke; a journal of cerebral circulation*. 2011
105. Janssen PM, Visser NA, Dorhout Mees SM, Klijn CJ, Algra A, Rinkel GJ. Comparison of telephone and face-to-face assessment of the modified rankin scale. *Cerebrovasc Dis*. 2010;29:137-139
106. McPhail S, Lane P, Russell T, Brauer SG, Urry S, Jasiewicz J, et al. Telephone reliability of the frenchay activity index and eq-5d amongst older adults. *Health Qual Life Outcomes*. 2009;7:48
107. Kosior JC, Idris S, Dowlatshahi D, Alzawahmah M, Eesa M, Sharma P, et al. Quantomo: Validation of a computer-assisted methodology for the volumetric analysis of intracerebral haemorrhage. *International journal of stroke : official journal of the International Stroke Society*. 2011;6:302-305

Table 1: Frequency of Ischemic Lesions after Intracerebral Hemorrhage

Study	N	Time to MRI	Patients with DWI Lesion	DWI Lesion Frequency (%)
**Gioia and Butcher**	54	2.5 days (median)	10	18.5%
**Menon et al[80]**	138	2 days (median)<br>35 days (median)	48<br>37	35%<br>27% (83% incidence) †
**Kang et al[85]**	97	3 days (median)	26	27%
**Arsava et al[86]**	86	14 days (maximum)	15	17%
**Garg et al[82]**	95	2 days (median)	39	41%
**Prabhakaran et al[84]**	118	1 day (median)	27	23%
**Gregoire et al[83]**	114	7 days (median)	16	14%
**Total***	**648**		**171**	**26%**

† Patients were scanned serially; 83% of lesions on second scan were not present on the first scan. Note, these 37 patients were not included in the totals to avoid duplication. *Total includes published data only (*Gioia and Butcher abstract submitted to International Stroke Congress, 2014*).

Table 2: Acute blood pressure treatment schedule for <140 mmHg Treatment Group.

Initial Treatment	
<140 mmHg Target Group	Target SBP < 140 mmHg within 30 minutes of randomization
Monitoring	• Continuous HR monitoring × 24 h minimum<br>• Record BP/HR q* 5 min during active treatment; q 15 min × 1 h, q 30 min × 5 h and q 1 h × 18 h
Labetalol (IV)	• Labetalol test dose: 10 mg bolus over 1 min<br>• If SBP ≥ 140 mmHg and HR > 55 BPM, repeat 10 mg bolus in 5 minutes.<br>• 10-20 mg IV push q 5 min until SBP < 140 mmHg or HR < 55 BPM<br>• Maximum labetalol dose: 300 mg / 24 h
And Enalapril (IV) (If available)	• Enalapril 1.25 mg bolus
And/or Hydralazine (IV)	If BP persistently > 140 mmHg:<br>• Hydralazine test dose: 5 mg IV bolus over 1 min<br>• If SBP ≥ 140 mmHg, repeat 5 mg IV bolus in 5 min

	• 10-20 mg IV bolus q 5 min until SBP < 140 mmHg<br>• Maximum hydralazine dose = 240 mg/24 h
Continuous IV Infusions (ICU admission)	If BP persistently >140 mmHg:<br>• Labetalol infusion 2–8 mg/min to a maximum of 300 mg/24 h and/or hydralazine infusion 50–150 µg/min
**Maintenance Therapy**	
Maintain SBP < 140 mmHg × 24 h minimum	
IV treatment prn	If SBP > 140 mmHg at any point:<br>• Labetalol (10-20 mg) / hydralazine (10–20 mg) boluses. BP and HR should then be recorded 5 and 15 minutes later<br>• Enalapril 1.25 mg may be repeated q 6 h if SBP >140 mmHg<br>• If SBP ≤ 135 mmHg or HR < 55 BPM, hold maintenance dose

* q = every or each
** prn = when necessary

<u>Table 3: Acute blood pressure treatment schedule for <180 mmHg Treatment Group.</u>

**Initial Treatment**	
<180 mmHg Target Group	Protocol to be used <u>only</u> if SBP ≥ 180 mmHg
Monitoring	● Continuous HR monitoring × 24 h minimum<br>● Record BP/HR q* 5 min during active treatment; q 15 min × 1 h, q 30 min × 5 h and q 1 h × 18 h
Labetalol (IV)	● Labetalol test dose: 10 mg bolus over 1 min<br>● If SBP ≥ 180 mmHg and HR > 55 BPM, repeat 10 mg bolus in 5 minutes.<br>● 10-20 mg IV push q 5 min until SBP < 180 mmHg or HR < 55 BPM<br>● Maximum labetalol dose: 300 mg / 24 h
Hydralazine (IV)	If BP persistently > 180 mmHg:<br>● Hydralazine test dose: 5 mg IV bolus over 1 min<br>● If SBP ≥ 180 mmHg, repeat 5 mg IV bolus in 5 min<br>● 10-20 mg IV bolus q 5 min until SBP < 180 mmHg<br>● Maximum hydralazine dose = 240 mg/24 h
**Maintenance Therapy**	
IV treatment prn	If SBP > 180 mmHg at any point:<br>● Labetalol (10-20 mg) / hydralazine (10–20 mg) boluses. BP and HR should then be recorded 5 and 15 minutes later

* q = every or each
** prn = when necessary

Appendix A: Summary of Trial Visits and Procedures

	VISIT 1	VISIT 2	VISIT 3	VISIT 4	VISIT 5	VISIT 6
	**Screening/ Randomization**	**24 hours (± 3 hours)**	**48 hours (± 12 hours)**	**Day 7 (± 2 days)**	**Day 30 (± 5 days)**	**Day 90 (± 30 days)**
Eligibility Criteria	X					
Signed Informed Consent	X					
Past Medical History	X					
Vital Signs (BP, HR)	X	X	X	X	X	X
Medications	X			X	X	X
Neurological Exam	X			X	X	X
**Blood Pressure Monitoring**	←	———	———	→		
**CT scan**	**X**	**X**				
**MRI scan**			**X**	**X**	X	
**NIHSS**	X		**X**	**X**	**X**	**X**
Glasgow Coma Scale (GCS)	X		X	X	X	X
Modified Rankin Scale (mRS)	X			X	X	X
Montreal Cognitive Assessment	X			X	X	X
Barthel Index					X	X
EuroQOL					X	X
SAE Reporting		X	X			
End of Study Report						X

Appendix B: Figures

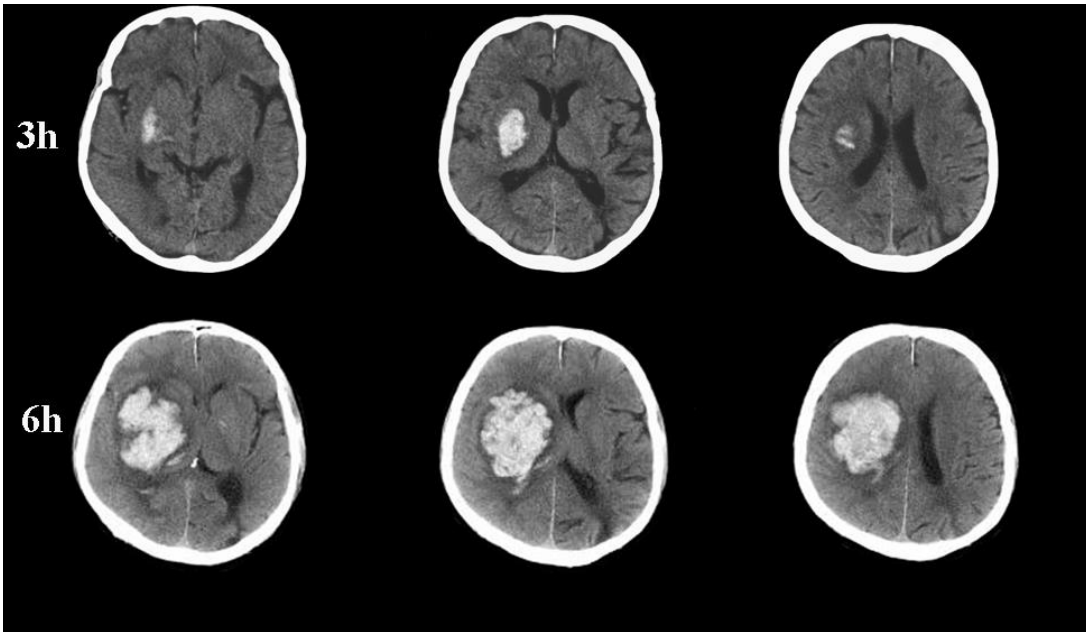

Figure 1. Example of fatal ICH (putamen) expansion in a 76 year old acutely hypertensive patient imaged 3 and 6 hours after symptom onset

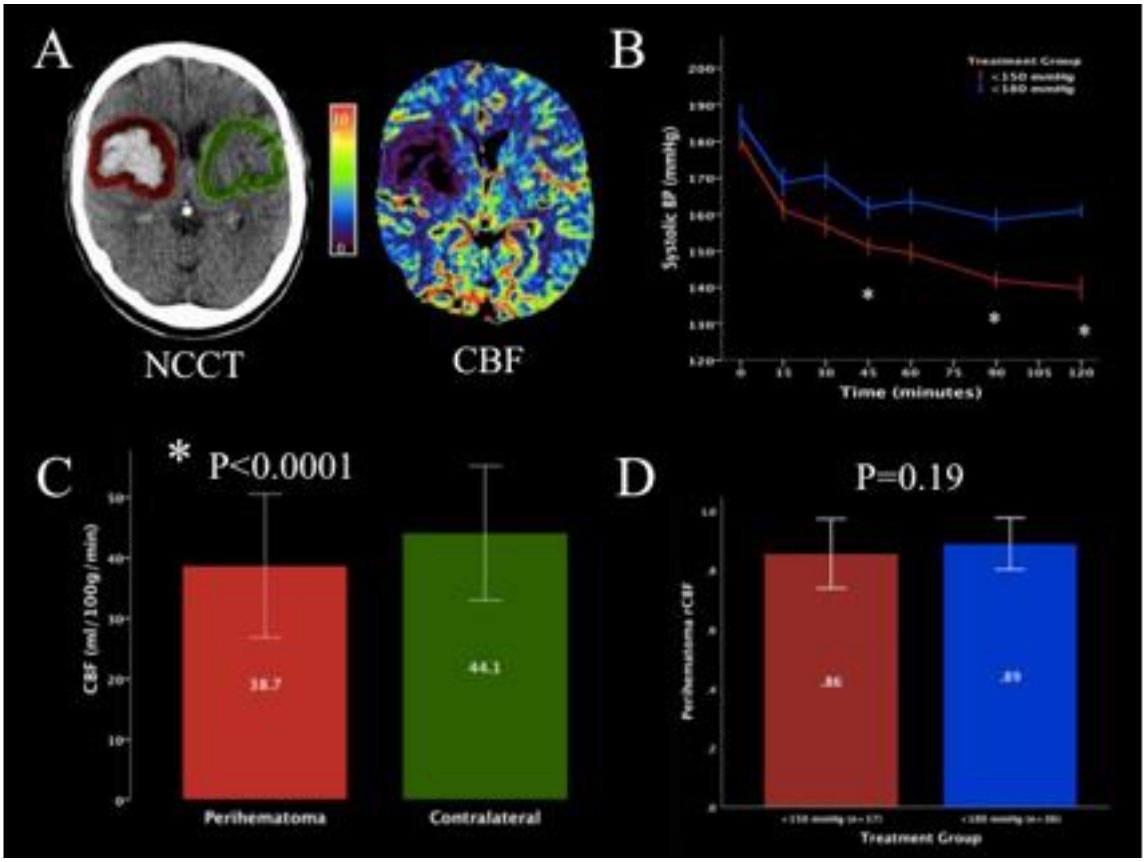

Figure 2. ICHADAPT I results summary. Perihematoma Cerebral Blood Flow (CBF) was measured in ICH patients (A). Patients were randomized to two different systolic blood pressure (BP) targets (B). Absolute perihematoma CBF was significantly lower than that in contralateral homologous regions in all patients (C). Relative CBF (rCBF) did not differ between two different BP treatment groups (D).

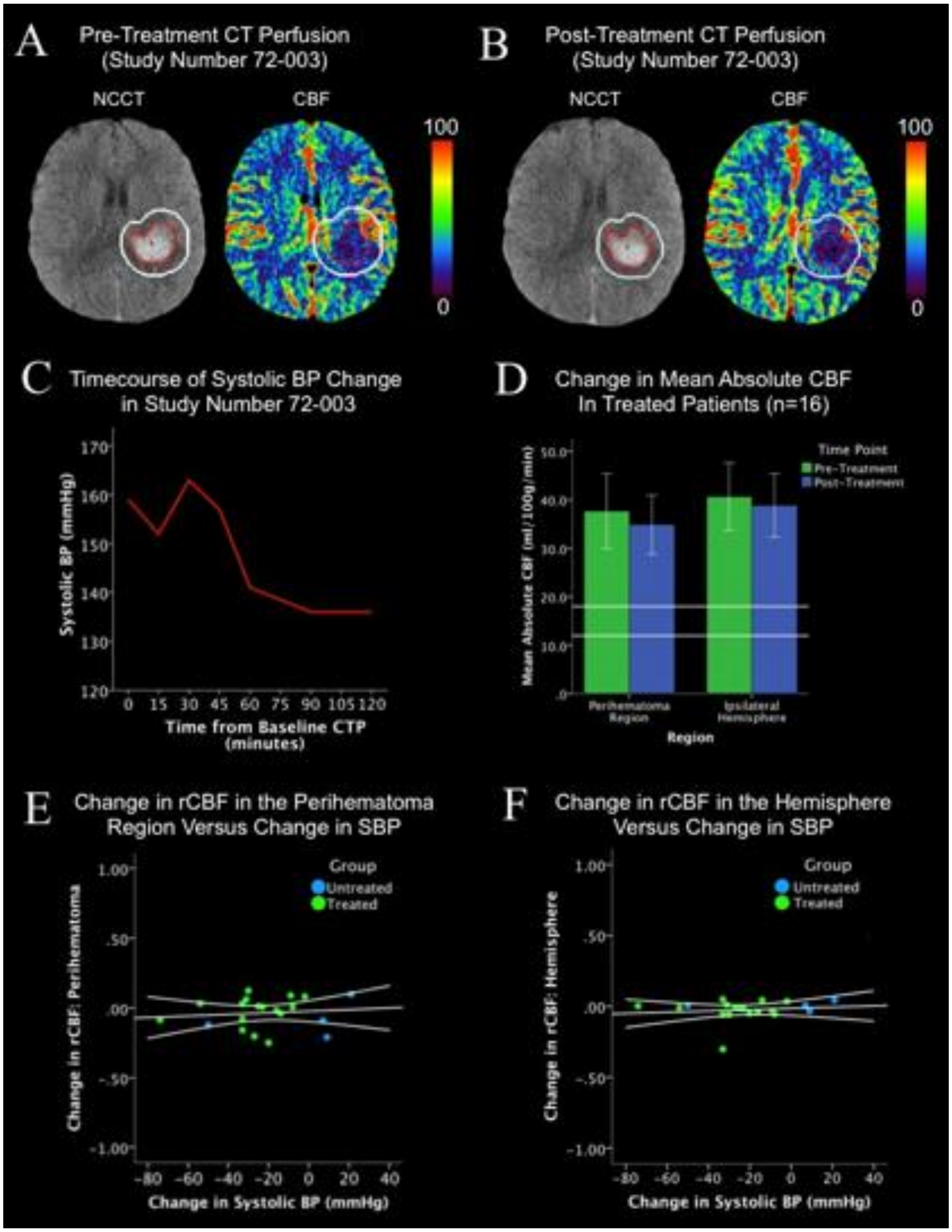

Figure 3. ICHADAPT I autoregulation sub-study summary. Non-contrast CT (NCCT) and Cerebral Blood Flow (CBF) measurements were repeated pre and post blood pressure (BP) reduction. Example of stable CBF in a 54 year old acutely hypertensive ICH patient treated with IV labetalol (A and B). Between the two time points show, hematoma (outlined in red) volume remained stable (15.1 ml to 15.3ml); Despite a SBP decrease from 159 mmHg to 136 mmHg (C), CBF remained stable throughout. Mean CBF in the perihematoma region and ipsilateral hemisphere did not change in patients treated with antihypertensive agents (D). There was no relationship between the change in BP and CBF in either the perihematoma region (E) or ipsilateral hemisphere (F).

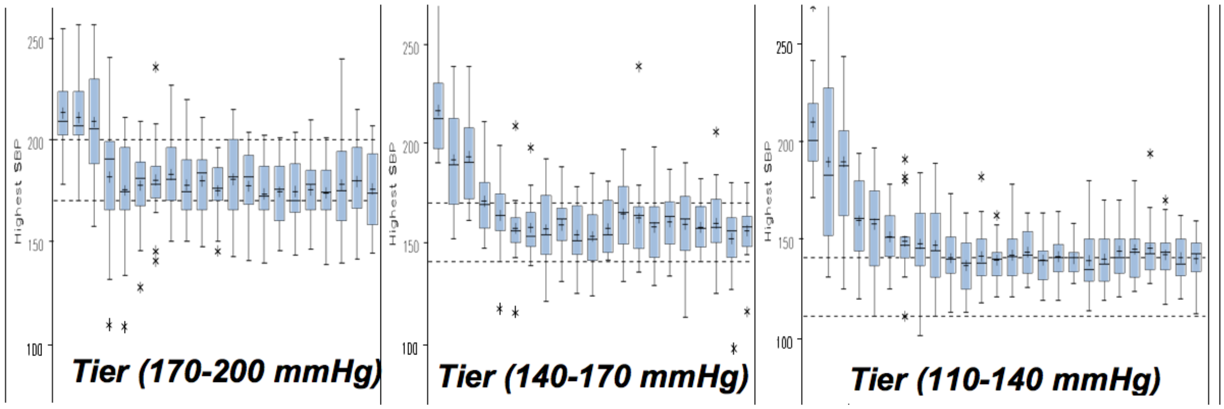

Figure 4. Effect of intravenous nicardipine infusions on systolic blood pressure reduction in ATACH I. Systolic Blood Pressure (SBP) measurements are taken every 15 min x 4, then every 30 min x 6 and then hourly. Nicardipine quickly lowered the SBP to the target range and maintained it there for 24 h.

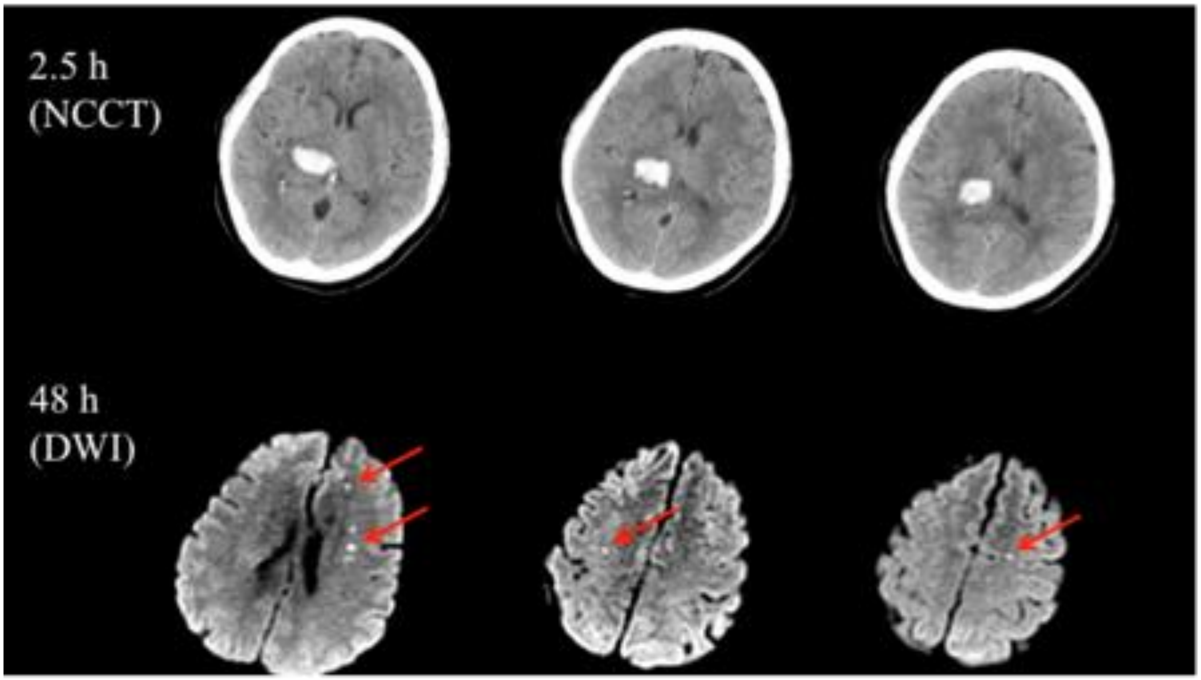

Figure 5. Non-contrast CT (NCCT) of acute hemorrhage and Diffusion-weighted MRI (DWI) lesions in subacute ICH patient (48 hours post symptom onset). Ischemic lesions (red arrows) are evident in the deep white matter (internal border zone territory) of both hemispheres (ipsilateral and contralateral to the hematoma). Systolic blood pressure was lowered from 200

mmHg to 160 mmHg acutely (nonrandomized treatment at direction of the attending physician).

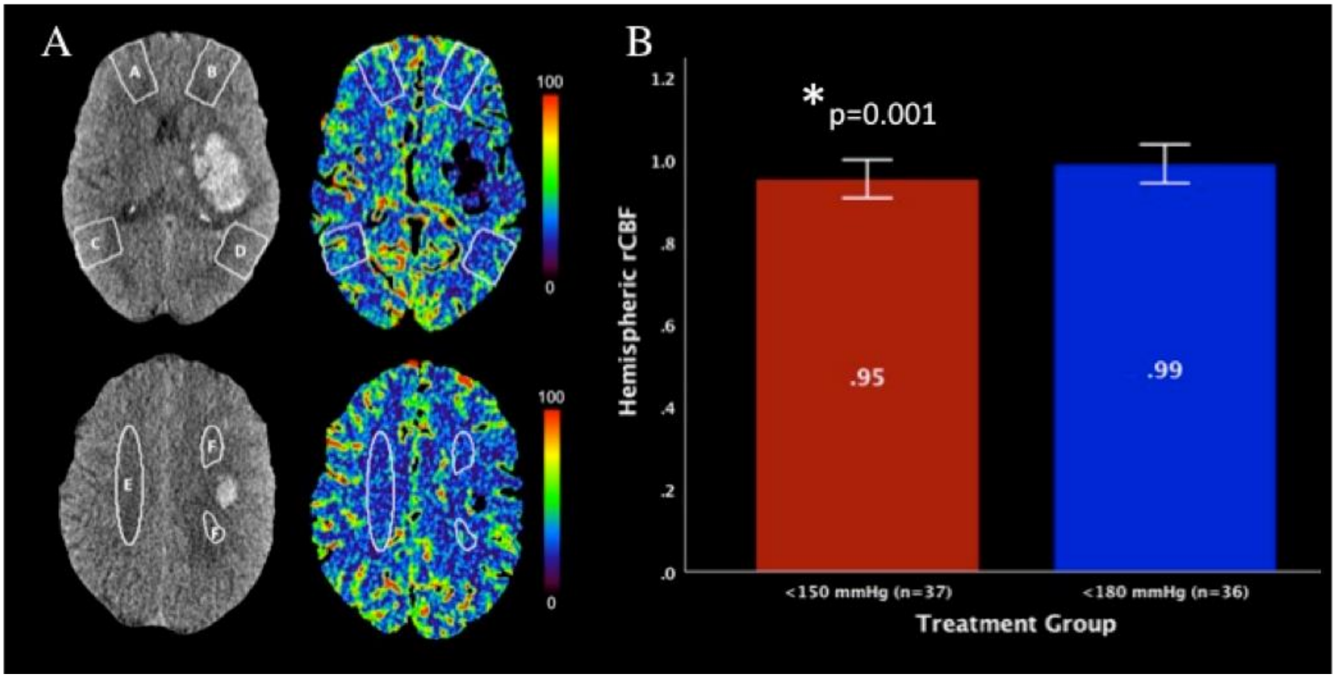

Figure 6. Hemispheric Cerebral Blood Flow (CBF) measurement in a patient randomized to a systolic BP target of <150 mmHg in ICH ADAPT I (A). The internal and external border zones between the Anterior/Middle/Posterior Cerebral Arteries are outlined. Hemispheric CBF was significantly lower in patients randomized to the <150 mmHg target (B), although exploratory analysis did not reveal a differential effect in the border zones.

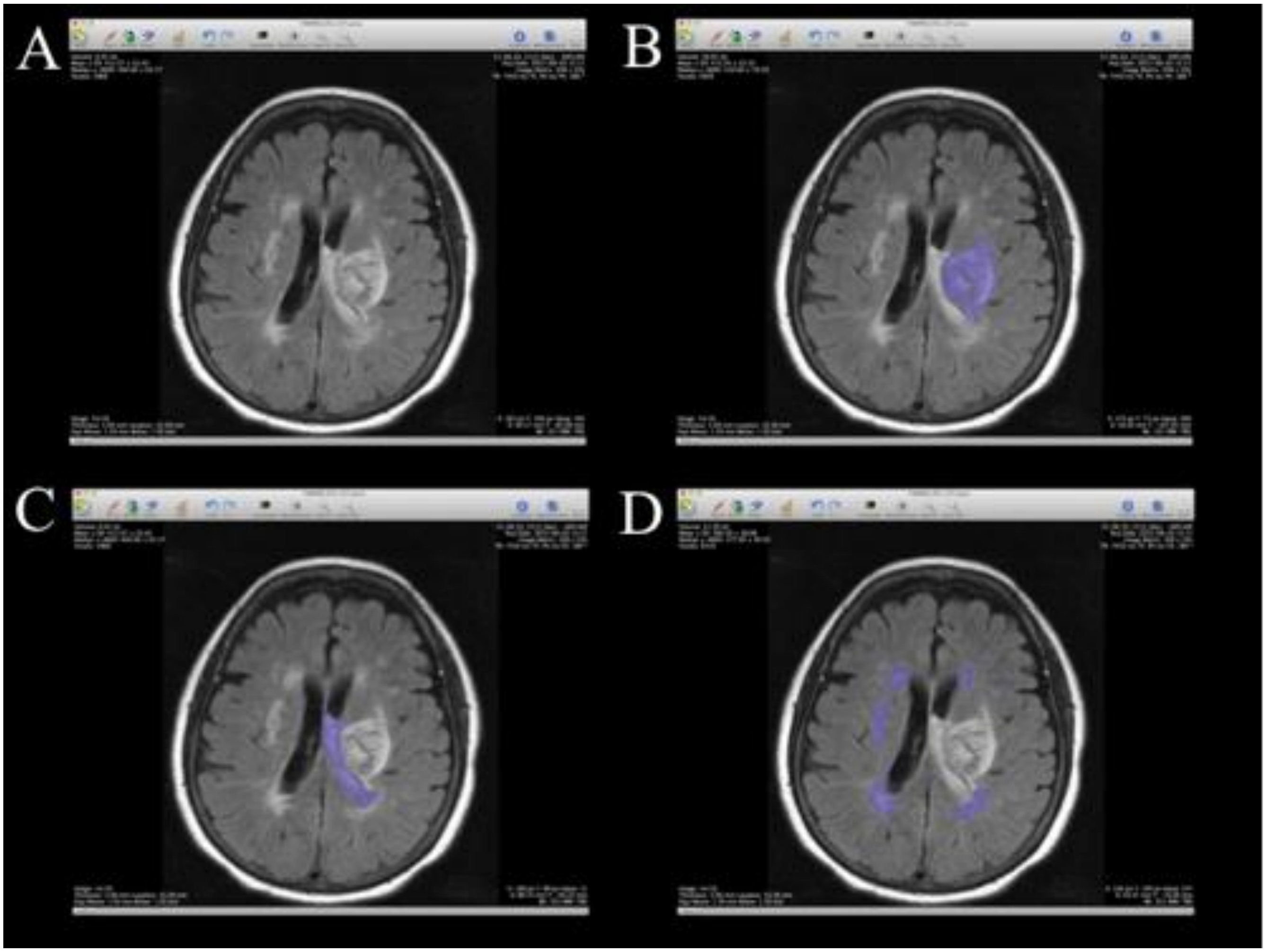

Figure 7. Segmentation and planimetric volume region of interest analysis using Quantomo software (Cybertrial, Inc.) in an MRI obtained in an ICH patient 46 h after symptom onset. Quantomo screenshots show MRI (FLAIR; Fluid Attenuated Inverse Recovery) (A) un-segmented image, (B) Hematoma (purple), (C) Periventricular and deep white matter hyper-intensities (Leukoariaiosis).
