# Supplementary material for: Acute Blood Pressure Lowering and Risk of Ischemic Lesions on MRI After Intracerebral Hemorrhage
Source: JAMA Neurol. 2025 Apr 21;82(6):543–50. doi: 10.1001/jamaneurol.2025.0586 (PMC12012699; doi:10.1001/jamaneurol.2025.0586)
Supplement: Supplement 2. — Statistical Analysis Plan [file jamaneurol-e250586-s002.pdf]

STATISTICAL ANALYSIS PLAN

Intracerebral Hemorrhage Acutely Decreasing Arterial Pressure (ICHADAPT) 2 Study

Feb 4, 2024

Version 1.0

SIGNATURES OF APPROVAL

Ken Bucher, MD, PhD, Principal Investigator
University of New South Wales

Feb. 5/2024
Date

February 5, 2024

Ashkan Shoamanesh, MD, Investigator
Population Health Research Institute

Date

LIST OF ABBREVIATIONS

AE	Adverse Event
AIS	Acute Ischemic Stroke
CI	Confidence Interval
DWI	Diffusion Weighted Imaging
ECG	Electrocardiogram
EMS	Emergency Medical Services
e-CRF	Electronic Case Report Form
GCS	Glasgow Coma Scale
ICH	Intracerebral Hemorrhage
ICHADAPT2	Intracerebral Hemorrhage Acutely Decreasing Arterial Pressure Trial 2
IQR	interquartile range
MRI	Magnetic Resonance Imaging
mRS	Modified Rankin Scale
NCCT	Non-contrast Computed Tomography Scan
NIHSS	National Institutes of Health Stroke Scale
PP	Per Protocol
SAE	Serious Adverse Event
SD	Standard Deviation

1.0 INTRODUCTION

This document provides the details of statistical analyses planned for the ICHADAPT2 study. In addition, it discusses the statistical issues relevant to these analyses (i.e. sample data to be used and missing data).

1.1 Background

The ICHADAPT2 study is a randomized trial in acute intracerebral hemorrhage (ICH) patients comparing a target systolic BP (SBP) of <140mmHg or <180mmHg within 6 hours of onset. The primary outcome is diffusion-weighted imaging (DWI) lesion incidence assessed on MRI 48±12 hours after randomization.

1.2 Primary Objective

The primary objective of the study is to determine whether aggressive systolic blood pressure reduction, to a target of <140 mmHg, is associated with an increase in the incidence of MRI signatures of acute cerebral ischemia (DWI lesions).

1.3. Study Design

Randomized, open label blinded evaluation trial comparing the effect of two different acute blood pressure targets on DWI lesion incidence 48±12 hours after randomization. Patients are randomized using permuted blocks of randomly varying sizes of 4, 6 or 8, stratified by hospital site, age, Glasgow Coman Scale, anticoagulant use, baseline ICH volume and intraventricular extension.

Inclusion Criteria:

1. Age ≥ 18 years
2. Two BP measurements ≥ 140 mmHg recorded >2 minutes apart
3. Acute primary ICH with consistent history and CT scan confirmation
4. Hematoma volume on CT must be <90 mL, as estimated using the ABC/2 method
5. Onset ≤6 hours prior to randomization
6. GCS ≥5 prior to randomization

Exclusion Criteria:

1. Known definite CONTRAINDICATION to the BP reduction protocol (i.e. severe arterial stenosis, Moyamoya disease or Takayasu's arteritis or high-grade stenotic valvular heart disease)
2. Known definite INDICATION for BP reduction (i.e. hypertensive encephalothopy or aortic dissection)

3. Known definite contraindication to MRI (i.e. cardiac pacemaker)
4. Definite evidence that the ICH is secondary to a structural brain abnormality (i.e. AVM, aneurysm, tumor, trauma or hemorrhagic transformation of ischemic infarct).
5. Previous ischemic stroke within 90 days of current event (NB: Prior ICH is not a contraindication)
6. Known history of intracranial neoplastic or vascular lesion
7. Subdural, subarachnoid or epidural hemorrhage
8. Planned resection of hematoma
9. Pre-existing disability and dependence (defined as pre-morbid Modified Rankin Scale Score ≥3)
10. Life expectancy <6 months due to pre-morbid conditions/terminal illness
11. Pervious participation in this trial
12. Current participation in another interventional trial

1.4. Sample Size Determination

A total of 270 participants will be randomized. This sample size reflects a 50% inflation of the required Evaluable Population (180) of patients undergoing MRI. Due to medical instability, approximately 50% of ICH patients are unable to undergo MRI, necessitating this inflation. An Evaluable Population of 180 participants provides 80% power to reject the null hypothesis that DWI lesion incidence rates are not associated with acute blood pressure reduction (one-sided alpha of 5%). This estimate is based on the following assumptions:

1. Equal allocation (1:1) to systolic BP targets of <140 mmHg and <180 mmHg and equal number of evaluable patients
2. An expected DWI lesion frequency of 26% in the <180 mmHg target group
3. A target systolic blood pressure of <140 mmHg will be associated with a 22% absolute increase in the frequency of DWI lesions

Table 1. Schedule of Assessments

	Screening/ Randomization	24±3 hours	48±12 hours	Day 7±2	Day 30±5	Day 90±30
Eligibility Criteria	☐					
Signed Informed Consent	☐					
Past Medical History	☐					
Vital Signs (BP, HR) Monitoring	☐	☐	☐	☐	☐	☐
Medications	☐			☐	☐	☐
CT scan	☐	☐				
MRI scan			☐	☐	☐	
NIHSS	☐	☐	☐	☐	☐	☐
Glasgow Coma Scale	☐	☐	☐	☐	☐	☐
Modified Rankin Scale	☐			☐	☐	☐
Barthel Index, EuroQOL					☐	☐
SAE Reporting		☐	☐	☐	☐	☐
End of Study Report						☐

1.5 Blinding

The study is conducted in an open-label blinded endpoint evaluation manner.

1.6. Definitions

Baseline: A subject's baseline value for a given endpoint or parameter is defined as his/her latest measurement taken prior to randomization.

Prior and Concomitant Medications: Prior medications are defined as those taken within three days of randomization. Concomitant medications are defined as those taken during study follow-up. All prior and all concomitant medications will be recorded on the REDCap electronic case report form (e-CRF).

2.0 ANALYSIS POPULATIONS

2.1 Intent-to-Treat Population

The study will be analysed by intention to treat, irrespective of actual blood pressure treatment or reduction achieved.

2.2 Evaluable Population (EP)

The EP population will include all randomized patients who undergo MRI with diffusion weighted imaging sequences at 48±12 hours.

3.0 INTERIM ANALYSES

There will be no interim analysis.

4.0 MISSING DATA AND DATA TRANSFORMATION

All efforts will be undertaken to avoid missing data. Missing vital sign data will be imputed using interpolation methods.

5.0 STATISTICAL METHODS

The software used for all summary statistical analyses will be Python (Python Software Foundation), with the NumPy, Statsmodels and SciPy libraries. Percentages will be rounded to one decimal place, except 0% and 100% will be displayed without any decimal places. Minima and maxima will be rounded to the precision of the original value; means and medians will be rounded to one decimal place greater than the precision of the original value; SDs will be rounded to two decimal places greater than the precision of the original value. P-values will be reported to four decimal places (0.xxxx), with values less than 0.0001 presented as <0.0001.

Inferential analyses will generally include statistics such as 2-sided 95% confidence intervals (CI), and p-values. The primary outcome will be assessed using a 1-sided statistical test. Unless stated otherwise, all other statistical tests will be 2-sided.

The primary outcome (Incident DWI lesion) will be analysed in the Evaluable Population by intention to treat.

5.1 Demographic and Baseline Characteristics

Subject demographic and baseline characteristics will be summarized with descriptive statistics for each treatment group. Demographic variables include but are not limited to age and sex.

Baseline characteristics include but are not limited to total intracerebral hemorrhage volume on diagnostic CT scan, past medical history and medications taken at the time of the stroke.

6.0 ANALYSIS

6.1.1 Primary Outcome

The primary outcome will be the incidence of acute DWI lesions on brain MRI obtained 48±12 hours after randomization.

Secondary Outcomes:

1. Number of DWI lesions on brain MRI 48±12 hours after randomization
2. Volume of DWI lesion on brain MRI 48±12 hours after randomization
3. Cumulative DWI lesion incidence on brain MRI 48±12 hours and 7±2 days after randomization

6.1.2 Statistical Hypothesis

The primary hypothesis is: the proportion of patients with DWI lesions in the <140 mmHg treatment arm will be >0.48.

6.1.3 Primary Analysis

The primary analysis will be a two sample, one-sided test of proportions at the alpha=0.025 level.

6.2. Secondary Analyses

1. The number of DWI lesions will be compared between the <140 mmHg and <180 mmHg treatment arms using a Mann-Whitney U test, as the distribution of lesion counts is expected to be non-normal.

2. The mean volume of DWI lesions will be compared between the treatment groups using an independent samples t-test if the data meet the assumption of normality. If the assumption of normality is violated, a non-parametric Mann-Whitney U test will be performed.

3. The cumulative incidence of DWI lesions within 7±2 days will be compared between the treatment groups with a two-sample, one-sided test of proportions at the alpha=0.025 level.
