# Supplementary material for: Acute Blood Pressure Lowering and Risk of Ischemic Lesions on MRI After Intracerebral Hemorrhage
Source: JAMA Neurol. 2025 Apr 21;82(6):543–50. doi: 10.1001/jamaneurol.2025.0586 (PMC12012699; doi:10.1001/jamaneurol.2025.0586)
Supplement: Supplement 3. — eTable. Univariable Predictors of DWI Lesions on 48-hour MRI in the Intracerebral Hemorrhage Acutely Decreasing Arterial Pressure 2 Trial (ICHADAPT-2) eFigure 1. Largest diffusion-weighted image (DWI) volume lesions on 48-hour MRI and more typical lesions in the Intensive Treatment Group (Target Systolic Blood Pressure <140 mm Hg; left) and the Conservative Treatment Group (Target Systolic Blood Pressure <180 mm Hg; right) in the Intracerebral Hemorrhage Acutely Decreasing Arterial Pressure 2 Trial eFigure 2. Study-level random-effects meta-analysis of the Intracerebral Hemorrhage Acutely Decreasing Arterial Pressure 2 Trial (ICHADAPT-2) and Antihypertensive Treatment of Acute Cerebral Hemorrhage II (ATACH-2) diffusion-weighted imaged (DWI) lesion secondary analysis [file jamaneurol-e250586-s003.pdf]

Supplemental Online Content

Butcher KS, Buck B, Dowlatshahi D, et al. Acute blood pressure lowering and risk of ischemic lesions on MRI after intracerebral hemorrhage. *JAMA Neurol.* Published online April 21, 2025. doi:10.1001/jamaneurol.2025.0586

eTable 1. Univariable Predictors of DWI Lesions on 48-hour MRI in the Intracerebral Hemorrhage Acutely Decreasing Arterial Pressure 2 Trial (ICHADAPT-2)
eFigure 1. Largest diffusion-weighted image (DWI) volume lesions on 48-hour MRI and more typical lesions in the Intensive Treatment Group (Target Systolic Blood Pressure <140 mm Hg; left) and the Conservative Treatment Group (Target Systolic Blood Pressure <180 mm Hg; right) in the Intracerebral Hemorrhage Acutely Decreasing Arterial Pressure 2 Trial
eFigure 2. Study-level random-effects meta-analysis of the Intracerebral Hemorrhage Acutely Decreasing Arterial Pressure 2 Trial (ICHADAPT-2) and Antihypertensive Treatment of Acute Cerebral Hemorrhage II (ATACH-2) diffusion-weighted imaged (DWI) lesion secondary analysis

This supplemental material has been provided by the authors to give readers additional information about their work.

© 2025 American Medical Association. All rights reserved, including those for text and data mining, AI training, and similar technologies.

eTable 1. Univariable Predictors of DWI Lesions on 48-hour MRI in the Intracerebral Hemorrhage Acutely Decreasing Arterial Pressure 2 Trial (ICHADAPT-2)

Predictor	Odds Ratio (95% CI)	P Value
Age	0.99 (0.96, 1.03)	0.7762
Sex	1.70 (0.66, 4.40)	0.2744
Previous ICH	0.68 (0.15, 3.07)	0.6133
Previous Ischaemic Stroke	1.19 (0.30, 4.22)	0.8692
Hypertension	0.89 (0.34, 2.35)	0.8104
Diabetes	0.82 (0.32, 2.12)	0.6876
Antiplatelet	1.20 (0.38, 3.75)	0.7539
Anticoagulant	0.77 (0.40, 1.47)	0.4261
Platelet Count	1.01 (0.998, 1.01)	0.1611
Glucose	0.89 (0.74, 1.08)	0.2476
Creatinine	1.002 (0.997, 1.008)	0.3148
Baseline GCS	0.94 (0.80, 1.11)	0.4724
Baseline NIHSS	1.01 (0.94, 1.09)	0.7210
Baseline Systolic BP	1.03 (1.00, 1.05)	**0.0151**
Systolic BP Change 1 hour	1.01 (0.99, 1.02)	0.5208
Systolic BP Change 24 hour	0.99 (0.97, 1.01)	0.2480
Weighted mean SBP 48 hours	1.04 (1.002, 1.08)	**0.0387**
Total ICH Volume	1.03 (1.01, 1.05)	**0.0060**
ICH Location (Lobar vs Deep)	3.96 (1.15, 13.65)	**0.0293**
Intraventricular Extension	1.32 (0.50, 3.52)	0.5744
Extra-axial Extension	1.83 (0.55, 6.14)	0.3235

ICH, intracerebral hemorrhage; GCS, Glasgow Coma Scale; NIHSS, National Institutes of Health Stroke Scale; BP, blood pressure.

© 2025 American Medical Association. All rights reserved, including those for text and data mining, AI training, and similar technologies.

eFigure 1. Largest diffusion-weighted image (DWI) volume lesions on 48-hour MRI and more typical lesions in the Intensive Treatment Group (Target Systolic Blood Pressure <140 mm Hg; left) and the Conservative Treatment Group (Target Systolic Blood Pressure <180 mm Hg; right) in the Intracerebral Hemorrhage Acutely Decreasing Arterial Pressure 2 Trial

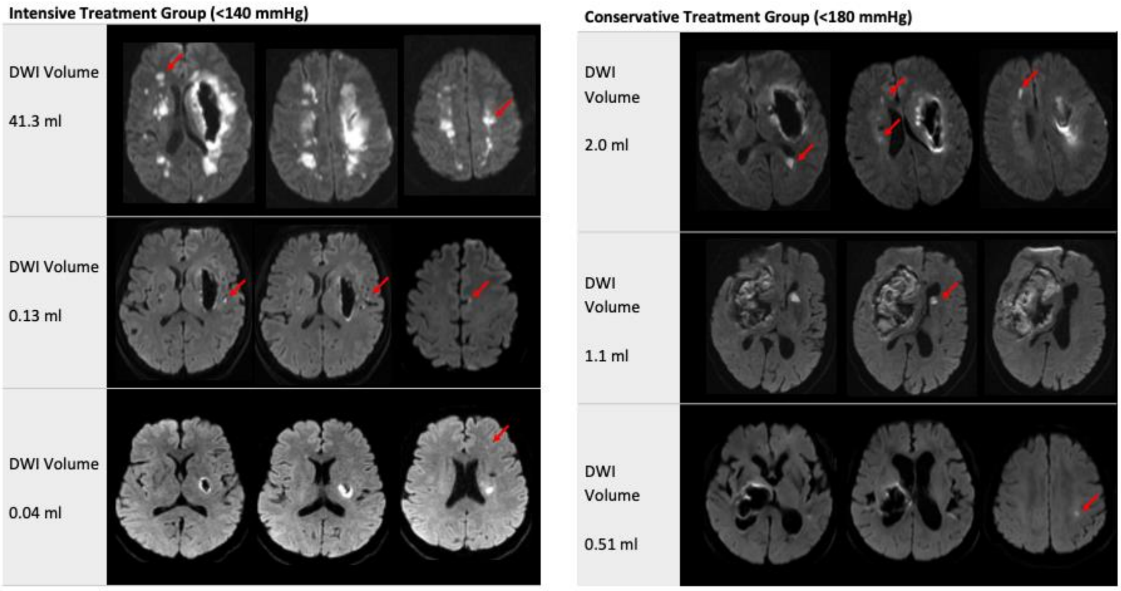

DWI volumes (mL) were measured using standard planimetric techniques.

© 2025 American Medical Association. All rights reserved, including those for text and data mining, AI training, and similar technologies.

eFigure 2. Study-level random-effects meta-analysis of the Intracerebral Hemorrhage Acutely Decreasing Arterial Pressure 2 Trial (ICHADAPT-2) and Antihypertensive Treatment of Acute Cerebral Hemorrhage II (ATACH-2) diffusion-weighted imaged (DWI) lesion secondary analysis

A.

Study or Subgroup	Experimental (Intensive) Events	Experimental (Intensive) Total	Control (Usual) Events	Control (Usual) Total	Weight	Risk Ratio M-H, Random, 95% CI
ICH ADAPT 2	13	42	14	37	42.4%	0.82 [0.44, 1.51]
ATACH 2	21	85	21	86	57.6%	1.01 [0.60, 1.71]
Total (95% CI)		127		123	100.0%	0.92 [0.62, 1.38]
Total events	34		35			

Heterogeneity: Tau² = 0.00; Chi² = 0.27, df = 1 (P = 0.60); I² = 0%
Test for overall effect: Z = 0.39 (P = 0.70)

Risk Ratio M-H, Random, 95% CI

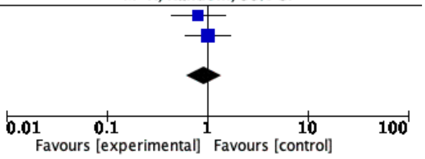

B.

Study or Subgroup	Experimental (Intensive) Events	Experimental (Intensive) Total	Control (Usual) Events	Control (Usual) Total	Weight	Risk Ratio M-H, Random, 95% CI
ICH ADAPT 2	16	49	18	46	48.6%	0.83 [0.49, 1.43]
ATACH 2	21	85	21	86	51.4%	1.01 [0.60, 1.71]
Total (95% CI)		134		132	100.0%	0.92 [0.63, 1.34]
Total events	37		39			

Heterogeneity: Tau² = 0.00; Chi² = 0.25, df = 1 (P = 0.62); I² = 0%
Test for overall effect: Z = 0.43 (P = 0.67)

Risk Ratio M-H, Random, 95% CI

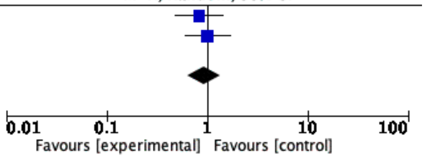

Patients in the ATACH-2 analysis underwent MRI between 1 and 10 days after onset. **A.** Risk ratios for DWI lesions in the combined 250 patients with acute ICH (including ICHADAPT-2 primary end point only; DWI at 48 hours) were not increased with intensive blood pressure reduction (experimental) versus conservative management (control). **B.** Risk ratios for DWI lesions in the combined 266 patients with an MRI within 7 days (ICHADAPT-2 secondary end point) were not increased with intensive blood pressure reduction (experimental) versus conservative management (control).

© 2025 American Medical Association. All rights reserved, including those for text and data mining, AI training, and similar technologies.
