# Supplementary material for: Acute Blood Pressure Lowering and Risk of Ischemic Lesions on MRI After Intracerebral Hemorrhage
Source: JAMA Neurol. 2025 Apr 21;82(6):543–50. doi: 10.1001/jamaneurol.2025.0586 (PMC12012699; doi:10.1001/jamaneurol.2025.0586)
Supplement: Supplement 4. — Data Sharing Statement [file jamaneurol-e250586-s004.pdf]

Data Sharing Statement

Butcher. Acute Blood Pressure Lowering and Risk of Ischemic Lesions on MRI After Intracerebral Hemorrhage. *JAMA Neurol*. Published April 21, 2025.
doi:10.1001/jamaneurol.2025.0586

Data
Additional Information: clinicaltrials.gov (NCT02281838)
Data available: No

Additional Information
Explanation for why data not available: Data available: Yes Data types: Deidentified participant data How to access data: Upon request with an approved proposal and signed data access agreement. Requests should be emailed to the corresponding author at ken.butcher@unsw.edu.au When available: With publication Supporting Documents Document types: None Additional Information Who can access the data: Researchers whose proposed use of the data has been approved Types of analyses: For any purpose supported by a research proposal. Mechanisms of data availability: After approval of a proposal and with a signed data access agreement.
